# Diversity in bread and durum wheat stigma morphology and linkage of increased stigma length to dwarfing gene *Rht14*

**DOI:** 10.1007/s00122-024-04663-4

**Published:** 2024-06-14

**Authors:** Margaret Pallotta, Takashi Okada, Stuart Roy, Allison Pearson, Ute Baumann, Ryan Whitford

**Affiliations:** 1 School of Agriculture, Food and Wine, University of Adelaide, Adelaide, SA Australia; 2 Present Address: School of Biomedicine, Faculty of Health and Medical Sciences, University of Adelaide, Adelaide, SA Australia; 3 Present Address: Grains Research and Development Corporation, Canberra, ACT Australia; 4 Present Address: Murdoch’s Centre for Crop and Food Innovation, State Agricultural Biotechnology Centre, Food Futures Institute, Murdoch University, Murdoch, WA 6150 Australia

## Abstract

**Key message:**

The dwarfing allele *Rht14* of durum wheat associates with greater stigma length, an important trait for hybrid breeding, whilst major dwarfing alleles *Rht-B1b* and *Rht-D1b* showed little to no effect.

**Abstract:**

Although much understudied in wheat, the stigma is a crucial component for attaining grain set, the fundamental basis for yield, particularly in hybrid production systems where successful grain set relies on wind-driven pollen dispersal by the male parent and effective pollen capture by the female parent. Females with long stigma that exsert early are thought to be advantageous. Using glasshouse-grown lines, we examined variation in Total Stigma Length (TSL) across diverse panels comprising 27 durum and 116 bread wheat genotypes. Contrasting genotypes were selected for population development and genetic analysis. Quantitative trait loci (QTL) analysis was performed on a durum F_2_ population and a bread wheat recombinant inbred line (RIL) population. Contrasting with studies of anther length, we found no large effect on TSL of the GA-insensitive semi-dwarfing genes *Rht-B1* and *Rht-D1* in either durum or bread wheat. However, in durum cultivar Italo, we identified a region on chromosome 6A which is robustly associated with larger TSL and contains the *Rht14* allele for reduced plant height, a trait that is favourable for female line development in hybrid systems. This dual effect locus explained 25.2 and 19.2% of TSL phenotypic variation in experiments across two growing seasons, with preliminary results suggesting this locus may increase TSL when transferred to bread wheat. In a bread wheat, RIL population minor QTL on 1A and 2A was indicated, but the strongest association was with *Ppd-B1*. Methods developed here, and the identification of a TSL-enhancing locus provides advances and further opportunities in the study of wheat stigma.

**Supplementary Information:**

The online version contains supplementary material available at 10.1007/s00122-024-04663-4.

## Introduction

The global significance of bread wheat (*Triticum aestivum* L.), and to a lesser extent durum wheat (*Triticum turgidum*), as food crops has led to demand for ever-increasing production and piqued interest in the first step of grain production; gamete fertilisation as a consequence of pollen receipt by stigma. In both self-pollination and cross-pollination systems, efficacy of fertilisation is crucial in determining yield, with characteristics of reproductive organs being clearly fundamental to this. However, whilst wheat anther studies are plentiful, stigma are remarkably understudied. The need for detailed information on the wheat female organ and its diversity has long been recognised; however, the labour-intensive nature of sample collection and the high-resolution equipment needed for precise evaluation has likely discouraged investment in this area.

Traditional wheat breeding systems currently remain prevalent, but hybrid breeding systems are a focus of effort worldwide due to their potential for generating high-yielding hybrids by harnessing heterosis through cross-fertilisation with male sterile plants (Whitford et al. 2013). In hybrid systems, specific plant architecture traits are deployed to overcome high costs of F_1_ grain production, a significant barrier to commercialisation. Plant height is one such trait because wheat pollen largely disperses downwards, and therefore, the female parent should be slightly shorter than the male parent to enhance opportunity for pollen capture. Whilst the green revolution provided a number of dwarfing alleles for reducing plant height, it is important to recognise that some of these alleles have additional effects on plant phenotype which could impact the success of wheat hybrids, which rely on outcrossing. The common GA-insensitive dwarfing alleles *Rht-B1b* and/or *Rht-D1b* have also been linked with shorter anthers and reduced anther extrusion (Lu et al. 2013; Okada et al. 2019, 2021; Skinnes et al. 2010). In contrast, the GA-sensitive dwarfing gene *Rht24* was found to have no effect on reduced anther extrusion in a bread wheat association panel study (Wurschum et al. 2018). The GA-sensitive durum wheat gene *Rht14*, thought to be a GA 2-oxidase (Duan et al. 2022; Vikhe et al. 2019), is present in a parent of our durum wheat mapping population. Recent work uncovered the identity of *Rht24* (Tian et al. 2022) and sequence comparison suggests it is allelic to *Rht14*. The effect of these specific loci and alleles on stigma traits is, to date, largely unexplored. Regarding reproductive organs, on the male side anther extrusion, anther length, pollen abundance and pollen longevity are important characteristics and have been investigated in numerous studies (Akel et al. 2019; He et al. 2016; Muqaddasi et al. 2017, 2019, 2016). On the female side, pollination efficacy is thought to depend largely upon degree of stigma exsertion and duration of stigma receptivity (De Vries 1971; Imrie 1966; Sade et al. 2022; Zeven 1968). Accurate large-scale determination of stigma receptivity duration remains difficult to achieve. However, measurement of stigma length, whilst arduous, is more achievable for mapping populations of effective size and was therefore the focus of this study.

In tetraploid and hexaploid wheats, as in tomato (Shang et al. 2021) and rice (Prahalada et al. 2021), domestication processes have resulted in selection for strong cleistogamy and high levels of self-pollination. As anthesis approaches, stigma and anthers are completely contained within the floret. Pollen-shed somewhat coincides with peak stigma receptivity (Gupta et al. 2015; Imrie 1966). Although florets partially and temporarily open at anthesis (Okada et al. 2017) and anthers may be partially or fully extruded beyond the floret, pollen is largely shed whilst the floret is mostly closed, maximising the potential for pollen-stigma interaction when the stigma is receptive. In wheat cultivars with longer stigma, the tips may be somewhat exerted at this time of temporary floret opening and high receptivity. If fertilisation is achieved the floret remains closed and the stigma atrophies, but if fertilisation is unsuccessful the carpel swells (Okada et al. 2017), the floret is forced open and the pair of stigma spread further apart, facilitating increased exposure to airborne pollen, whether that be from the same plant or a neighbouring plant. Typically, the two stigma of wheat pistils are close to equal in length and in male sterile lines, after carpel swelling, stigma tips exsert out each side of the floret between the palea and lemma. In wheat, evidence that longer stigma lead to enhanced exertion and improved cross-pollination success is lacking, but support for the notion can be found in rice studies. Strong correlations were found between stigma length, stigma exsertion and grain set in a study of field-grown male sterile rice (El-Namaky 2018). Another study involving an association panel of 533 field-grown landraces and elite rice lines, ﻿Zhou et al. (2017) reported positive Pearson’s correlation coefficients for stigma exsertion with both stigma length and style length.

Trait heritability is a key factor in determining amenability for implementation in breeding programs, and estimates of wheat stigma length heritability have been attempted. In a study involving a diverse panel of 400 field-grown spring wheats which included exotic as well as adapted germplasm, Singh et al. (2007) reported a broad sense heritability value of 0.87 for stigma length. In contrast, a study of chemically induced male sterile field-grown winter bread wheats by Sade et al. (2022) found low heritability estimates (0.24–0.45) for stigma extrusion. In addition, a genome-wide association study of 196 field- or polytunnel-grown elite spring bread wheats by El Hanafi et al. (2021) found low heritability for stigma length. Their study identified just two minor QTLs, one on 1DL and the other on 2AL, explaining 8.0% and 9.55% of trait variation, respectively. To our knowledge, the bread wheat studies mentioned here are the only stigma diversity analyses described to date. No studies of stigma diversity have yet been reported for durum wheat.

Amongst other major crops in which stigma length or exsertion have been studied, rice is the most closely related to wheat, although rice pistils typically comprise longer styles and shorter stigmas than wheat. Variation in either of these rice pistil components may increase stigma exsertion outside the floret. Based on their GWAS study of 533 diverse *Oryza sativa* accessions, Zhou et al. (2017) found that grain-size genes *GW2*, *GS3* and *GW5* affect stigma exsertion. They proposed that *GW5* improves stigma exsertion in both *O. indica* and *O. japonica* as a secondary effect from the reduction in glume width. The *GW2* association, which was only evident in the *O. japonica* subset of the panel, was not further studied in detail. In the *O. indica* subpopulation, *GS3* increased exsertion by increasing style length. In an earlier *Oryza japonica* study by Takano-Kai et al (2011), a nonsense mutation in *GS3* was reported to increase stigma length. Dang et al. (2022) identified *SYL3* (LOC_Os03g14850) as a MADS-box family transcription factor that plays a role in determining style length in *Oryza sativa*. Allele *SYL-k* from cultivar Kasalath gave longer styles as a consequence of higher *OsGA3ox2* expression and GA_4_ content in pistils which leads to greater style cell length (Dang et al. 2022). They observed that allele *SYL-k* enhanced cross-pollination grain set on male steriles by 16%.

Exotic germplasm has frequently been a source of useful trait variants for use in breeding programs. Prahalada et al. (2021) describe an *Oryza longistaminata* locus on the long arm of chromosome 8 that was associated with increased stigma length and stigma width, as well as style length and style width. They report success in introgressing this locus into two elite cytoplasmic male sterile lines, leading to 2.5 to threefold higher grain setting rate in test crosses. Their study utilised simple sequence repeats (SSRs) as molecular markers, which makes syntenic extrapolation to wheat difficult. In bread wheat, there are numerous cases of trait improvement through introgression of exotic genome, as well as through transfer from more closely related species, an example being increased tolerance to high soil boron derived from a tetraploid land-variety (Pallotta et al. 2014).

The paucity of genetic studies in wheat combined with a lack of obvious targets from rice research encouraged us to undertake exploration of variation in stigma length in both durum and bread wheat. We aimed to establish a method for relevant, accurate and efficient measurement of stigma length, examine germplasm panels for diversity and identify lines with extreme stigma phenotypes for population development and genetic analysis. By QTL analysis of genetic mapping populations of both durum and bread wheat, and genetic association tests in a bread wheat diversity panel, we investigated the specific impact of dwarfing genes *Rht-B1b*, *Rht-D1b* and *Rht14* on TSL. Our findings revealed that *Rht-B1b* and *Rht-D1b* exhibit little to no effect on TSL in wheat. Notably, we identified a medium to large effect associated with the *Rht14* locus in durum wheat and demonstrated that it is transferable to bread wheat. This study provides valuable insights into the genetic factors influencing TSL in both durum and bread wheat.

## Methods

### Plant materials

#### Diverse bread wheat and durum wheat panels

Details of the panel of 116 spring bread wheat lines that were used for the initial screening are provided in Table S1, whilst details of the 27 durum wheats screened are given in Table S2. All grains used for the panels were from stocks maintained at the University of Adelaide. Stocks of *Ms3*/7*1IBWSN50 and *Ms2*/6*SUN276A were obtained from the Australian Grains Genebank, Horsham, Victoria (AGG).

#### BC_2_F_1_ backcross plants

*Ms2*/6*SUN276A lines that were heterozygous for the dominant *Ms2* male sterility gene (and therefore sterile) were used as female parents in crosses performed to initiate backcrossing of the *Rht14* locus from Italo into bread wheat. To more rapidly regain a full hexaploid complement, subsequent backcrosses were made by using selected fertile (*ms2ms2*) F_1_ plants as pollen donors onto sterile *Ms2ms2* plants of *Ms2*/6*SUN276A.

#### F_2_ bread wheat and durum wheat populations

F_2_ populations comprising 270 Mexicali/Italo durum wheat lines and 82 Thori/Hydra bread wheat lines were developed in this study from crosses between plants grown in the screening panel experiments, with Mexicali and Thori being female parents.

#### Mocho/Gladius RIL population

An F_2_ mapping population for Gladius/Mocho de Espiga Branca had previously been derived from a biparental cross between the Australian commercial cultivar Gladius (doubled haploid (DH)-derived Rac875/Krichuaff//Excalibur/Kukri/3/Rec875/Krichauff/4/-RAC875//Excalibur/Kukri) and Mocho de Espiga Branca, a Portuguese landrace (Borjigin et al. 2021). To develop a recombinant inbred line (RIL) mapping population, 1800 F_2_ grains were taken through single seed descent for four generations to develop F_6_ RILs. To reduce the time for population development, a modification of a speed breeding approach was used. Wheat lines were grown in soil in a growth room, located at the Waite Precinct, Urrbrae, South Australia, for approximately 12 weeks, under a photoperiod of 20 h light and 4 h dark. Temperatures ranged from 25 °C (day) to 15 °C (night). Grains from the plants were removed 15–18 days post anthesis. Embryos were removed from the grains and transferred to culture media to promote growth of the embryo. When the new wheat plantlet was large enough, it was transplanted into soil and placed back in the growth room, with the same conditions as above. The process was repeated for 4 generations. From the initial starting material of 1,800 F_2_ plants, 1,199 F_6_ RILs were produced. Of these lines, 320 were genotyped for linkage map generation and trait association studies.

### Growth conditions

With the exception of the Mocho/Gladius RILs, all plants were grown in pots containing either South Australian Research and Development Institute (SARDI) Urrbrae 6 mm Premium mix from BioGro (https://biogro.com.au/) or SARDI Coco-Peat mix and were grown in the same glasshouse at the University of Adelaide with temperatures ranging from approximately 15 °C (night) to 25 °C (day) and a 12–14 h photoperiod. Natural light was supplemented with halogen lights when necessary. Plants of the durum and bread wheat panels were grown singly in 4.5 L 200 mm round pots. We examined Mexicali/Italo F_2_ plants in two separate spring season glasshouse experiments, Expt-D1 and Expt-D2 involving 90 and 180 randomly selected F_2_ plants, respectively, along with parental controls. In the first (smaller) experiment, we found lines all flowered within a 10-day interval. Therefore, to manage sampling and imaging workload, the second experiment was sown in two parts with a two-week interval between sowing dates. Each of the groups comprised 90 F_2_ plants plus controls, and their respective flowering windows were 10 and 12 days. For all experiments, plants were grown singly in 0.8 L 90 mm square pots. This was also the case for the experiment involving Thori/Hydra F_2_ and parental controls.

For the Mocho/Gladius RIL population experiment, single plants of 210 genotyped RILs and three plants of each parental line were grown individually in round pots containing 2300 g soil mix (50% (v/v) University of California mic, 35% (v/v) peat mix and 15% (v/v) clay loam) in a glasshouse at The Plant Accelerator®, Waite Campus.

### Phenotyping

**Fig. 1:**
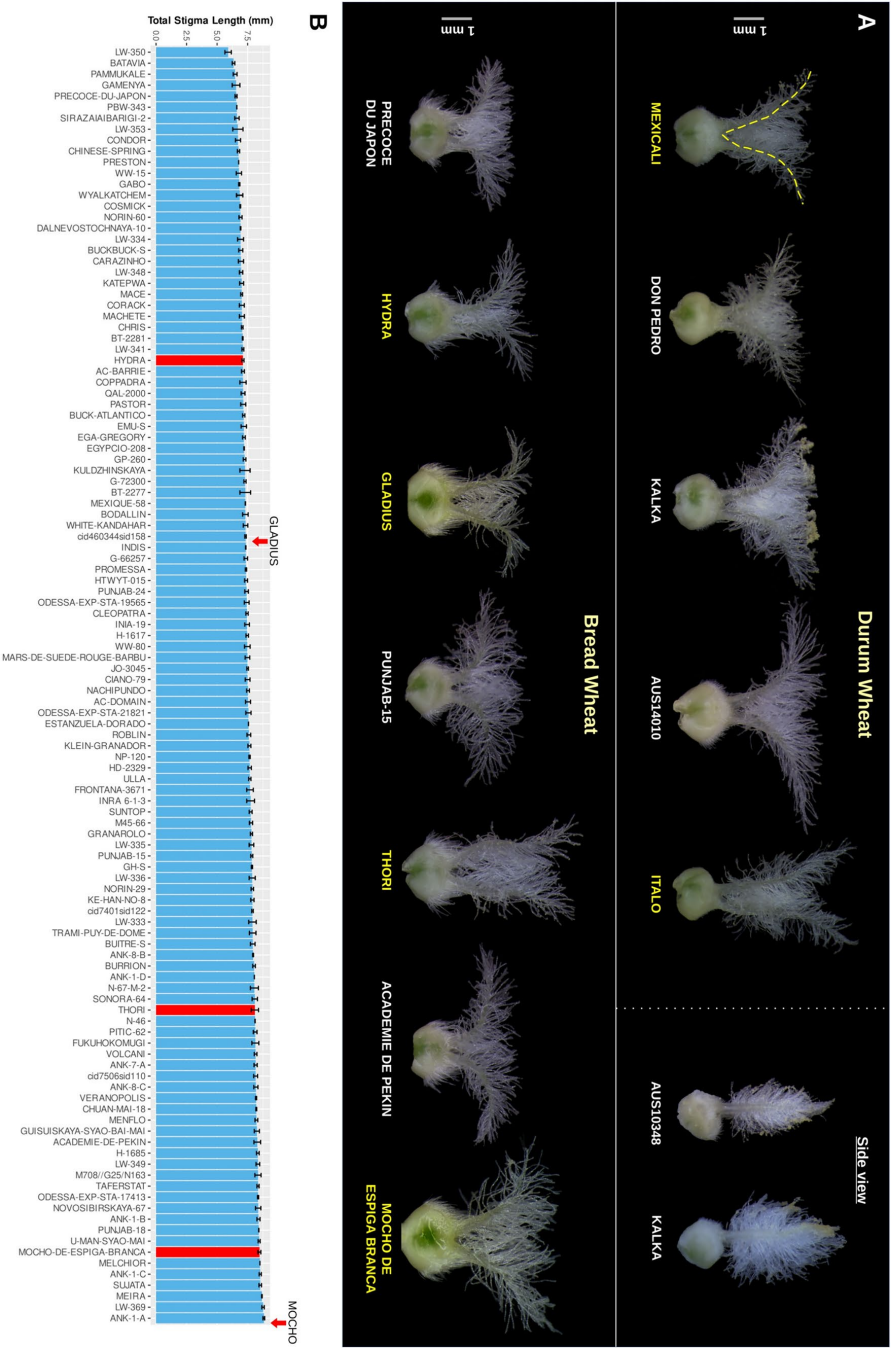
Diversity of stigma phenotype in durum and bread wheat. **A** Example carpel and stigma images of durum (upper) and bread (lower) wheat used for TSL measurement. The yellow dashed line indicates the manual tracing used within imaging software Fiji (https://fiji.sc/) for TSL calculation. The separate box in the upper row shows example side-view images of durum wheat stigma. **B** Distribution of TSL in a diverse panel of 116 bread wheats (Supplementary Table S1) grown in a glasshouse. The Y-axis indicates average TSL and error bars represent standard deviation. Along the X-axis lines are ordered from lesser (left) to higher (right) TSL. Red bars indicate lines used for developing genetic mapping populations. TSL of Gladius and Mocho in Expt-D2 (Table 1) is indicated by arrows above the plot

We measured stigmas from freshly sampled primary florets of main spikes. To ensure that we were measuring stigmas in their peak period of growth, we observed and developmentally staged plants daily, collecting florets that contained yellowing but not dehisced anthers. Generally, anthers in such florets dehisced at about the time of imaging. We collected two to three florets from each side of the central region of the main spike, giving a total of four to six pistils per line for imaging. To not damage pistils, florets were grasped at their distal end with blunt tweezers and gently detached from the rachis by a downward motion. All florets from a line were promptly placed into a single plastic 2 ml centrifuge tube. Tubes were immediately closed to maintain humidity and prevent stigma dehydration. After samples were collected, tubes were directly taken to the laboratory. Under a Leica MZFL III stereo dissecting microscope, working on one or two florets at a time, pistils were carefully removed and positioned on a dark plate (to allow image contrast), on a small knob of blackened Bostik BluTack®, for imaging such that the full breath of the stigma was in focus. Example images are provided in Fig. 1. Carpel hairs of bread wheat pistils were removed using fine tweezers so that unobscured images of both style branches and their junctions with the carpel were obtained. The only exception to this methodology was that for the bread wheat panel experiment carpel hairs were not removed before imaging. Carpel hair removal was usually not necessary for durum wheats because, as shown in Fig. 1, they typically had few and short carpel hairs. Images were captured with a Leica DFC300 digital camera. Tracing and measurement of Total Stigma Length (TSL) were performed manually for each image using image software Fiji (https://fiji.sc/) (Schindelin et al. 2012). For analysis, if stigmas of more than four pistils were measured, we used the top four TSL values. We did this because the most relevant information for this study was the maximum TSL achieved by a specific genotype, and we had noted that florets at sub-optimal development stages gave unrepresentative smaller TSL. On rare occasion, sampled florets in their 2 ml centrifuge tube were stored overnight at 4 °C for imaging the following day. Stigmas stored overnight at this temperature were observed to be indistinguishable from freshly harvested stigmas.

Plant Height (PH) was measured from soil surface to the top of the tallest spike at maturity. Days to Anthesis (DA) was calculated as the number of days from sowing until the onset of anthesis in the main spike (GS61 on the Zadoks scale). Peduncle Length (PL) was measured in Expt-D1 at anthesis as the length of the main stem from flag leaf ligule to the base of the spike. In Expt-D2 PL was similarly measured as the length of the main stem from flag leaf ligule to the base of the spike, however, for improved representation, measurement was at physiological maturity and an average PL was calculated from the PL of the first three culms.

### Genotyping

DNA was obtained from leaf tissue by either phenol:chloroform:IAA extraction (durum and bread wheat panels and parental lines) or rapid extraction from pulverised freeze-dried samples in 96-well plate format (F_2_ and RIL populations and parental controls). Phenol:chloroform:IAA extraction was performed as described (Pallotta et al. 2000). For 96-well rapid extraction, we used a protocol based on that initially reported in (Pallotta et al. 2003) and here more fully described in Supplementary methods.

#### Mexicali/Italo F_2_ population

Targeted genotyping of the Mexicali/Italo F_2_ population used KASP™ markers developed in this study based on parental SNP identification in DArTseq® data that was kindly provided by Prof. Dianne Mather, University of Adelaide. Further KASP™ markers were sourced from a local University of Adelaide collection. Although a *Rht14* causative mutation has yet to be identified, we sourced closely linked flanking markers cs214 and cs412 reported by (Ford et al. 2018) to track *Rht14* in our Mexicali/Italo population. In all instances, genotyping of *Rht-B1* and *Rht-D1* alleles used KASP™ markers (Rasheed et al. 2016). Durum marker sequences were aligned by BLAST at Ensembl Plants (https://plants.ensembl.org/index.html) to the *Triticum turgidum* reference sequence assembly GCA_900231445.1, also known as Svevo.v1 (Maccaferri et al. 2019).

#### Mocho/Gladius RIL population

Genotype information for Mocho/Gladius RIL genetic map development was obtained through targeted genotyping-by-sequencing (tGBS) performed by the Department of Primary Industries Victoria. Gladius and Mocho de Espiga Branca parents were also genotyped using a 90 K iSelect wheat array and wheat exome tGBS array. Genotypes for the RIL population were called using a custom genotype calling algorithm developed by the Department of Primary Industries Victoria, resulting in a total of 3493 polymorphic markers being identified. After exclusion of markers showing significant segregation distortion or low quality (> 60% missing values), we obtained a final set of 2774 markers whose sequence and SNP information is provided in Table S3. The set was supplemented by additional KASP™ markers for the phenology-related genes *Glu-A1*, *Ppd-B1* and *Vrn-A1* which were also kindly provided by Prof. Dianne Mather. Marker sequences were aligned by BLAST at Ensembl Plants (https://plants.ensembl.org/index.html) to the *Triticum aestivum* cultivar Chinese Spring reference sequence assembly GCA_900519105.1, also known as IWGSC REFSEQv1 (International Wheat Genome Sequencing Consortium 2018).

Assays for all KASP™ markers were performed according to manufacturer’s instruction using KASP™ mix reagent. Sequence information for all KASP™ markers used in this study is provided in Table S4.

### Statistical and QTL analyses

For QTL analyses of Mexicali/Italo F_2_ and Mocho/Gladius RIL populations draft, genetic linkage maps were constructed at LOD = 6 using the R package ASMap. Samples with high (> 20%) HET, low call rate (< 40%), high number of crossovers (based on non-imputed genotypes) and identical samples were removed. In total, for the Mocho/Gladius RIL population, 2778 markers from 309 RILs were used to generate a genetic linkage map. For Mexicali/Italo Expt-D1 and Expt-D2, 37 markers from 90 F_2_ plants and 17 markers from 180 F_2_ plants, respectively, were used to construct genetic linkage maps for targeted genomic regions involving chromosomes 4B, 6A and 6B only. QTL analyses were performed using “scanone”, “scantwo”, “makeqtl”, “refineqtl” and “fitqtl” functions of the R/QTL package (Broman et al. 2003). LOD score thresholds for each trait were determined by permutation test and only QTLs with LOD score above such thresholds are reported in this study. The percentage of phenotypic variation explained by the most significant marker under each identified QTL was calculated. For Mexicali/Italo Expt-D2, sowing date was used as a covariate for QTL analysis as it had a significant effect on traits (Sup Fig. S4). We used RStudio (RStudio Team 2020) to produce summary statistical data and graphs (bar plots, boxplots, scatter plots, and histograms), and to perform post-hoc Tukey’s tests or Student’s t tests for marker-trait associations and Pearson’s correlation analyses.

### Analysis of meiotic pairing at Metaphase I

Based on spike size and position below the penultimate leaf, immature spikes that were predicted to contain meiocytes at Metaphase I stage were collected by cutting stems and placing them immediately in water for transportation to the laboratory. Single anthers were excised under a binocular dissecting microscope, placed on a glass slide in a drop of 45% acetocarmine stain, gently squashed under a glass coverslip and then examined using a compound microscope and 10 × lens for meiotic stage. If the examined anther was at Metaphase I (M-I), this is indicative of the remaining two anthers from the floret being at the same stage. These were therefore excised and placed into a microcentrifuge tube containing freshly prepared fixative that comprised 3:1 ethanol: glacial acetic acid. Fixed anthers were stored overnight at 4 °C. The following day anthers were Feulgen-stained by treatment with 1N HCl at 60 °C for 8 min and then replacement by Schiff’s reagent. When anthers were well stained, generally after approximately 30 min, anthers were mounted on glass microscope slides in 45% acetocarmine, and a coverslip placed over the anther with another positioned under one edge of the first coverslip. Gentle tapping on the top coverslip using the back end of a pencil, or similar, whilst slowly removing the lower coverslip dispersed the anther tissue across the area beneath the coverslip. The slide was gently heated on a hotplate to draw down the coverslip, and then, the sample was firmly squashed between sheets of Whatman No. 1 filter paper. Prepared slides were examined for intact M-I cells and chromosome pairing counts made under 40× or 100× objectives.

## Results

### Stigma phenotype in durum and bread wheats

In our initial evaluation of variability in stigma presentation using our bread wheat panel of 116 diverse lines, we sampled and imaged stigmas from both primary and secondary florets within central spikelets. From these images, it was clear that stigma length differed markedly between the two florets of the same spikelet, even when the primary floret was at or very near anthesis. We deduced that the stigma grows rapidly as anthesis approaches and to reduce error we therefore standardised our sampling to primary florets only. We also standardised our sampling to spikes of main tillers only. When such spikes were at near anthesis, we generally found that the six central spikelets had primary florets at similar developmental stages and that their stigmas were optimal for imaging and subsequent measurement. In the durum wheats examined, we noted that stigmas atrophy more rapidly than bread wheats upon pollen shed and therefore collected samples in smaller batches, whilst also ensuring that they were on the cusp of anther dehiscence.

The top portion of carpels of all bread wheats examined were densely covered with long hairs that require partial removal for accurate measurement of stigma length, as exemplified in Fig. 1. The additional time required for carpel hair removal in bread wheats unfortunately made these experiments more laborious. In contrast, carpels for all but three of our durum wheat panel of 27 diverse lines had considerably shorter hairs that did not require removal before imaging. The three exceptions, Khapli, AUS-10105 and Morocco-W-74633, had long carpel hairs that were similar to those observed for the bread wheats. The cultivar Don Pedro was found to have an essentially hairless carpel (Fig. 1). In both panels, we observed substantial variation in stigma length, stigma hair density and stigma hair length. We did not observe notable differences in the distance between the pair of style branches at their carpel junctions. Of the obviously variable traits, stigma length was deemed to be the most amenable for accurate measurement. To reflect the total breadth of stigma available for pollen capture, our measurement, Total Stigma Length (TSL), is the total length from stigma tip to stigma tip, including style and carpel tissue between style branches, as shown in Fig. 1A.

**Fig. 2:**
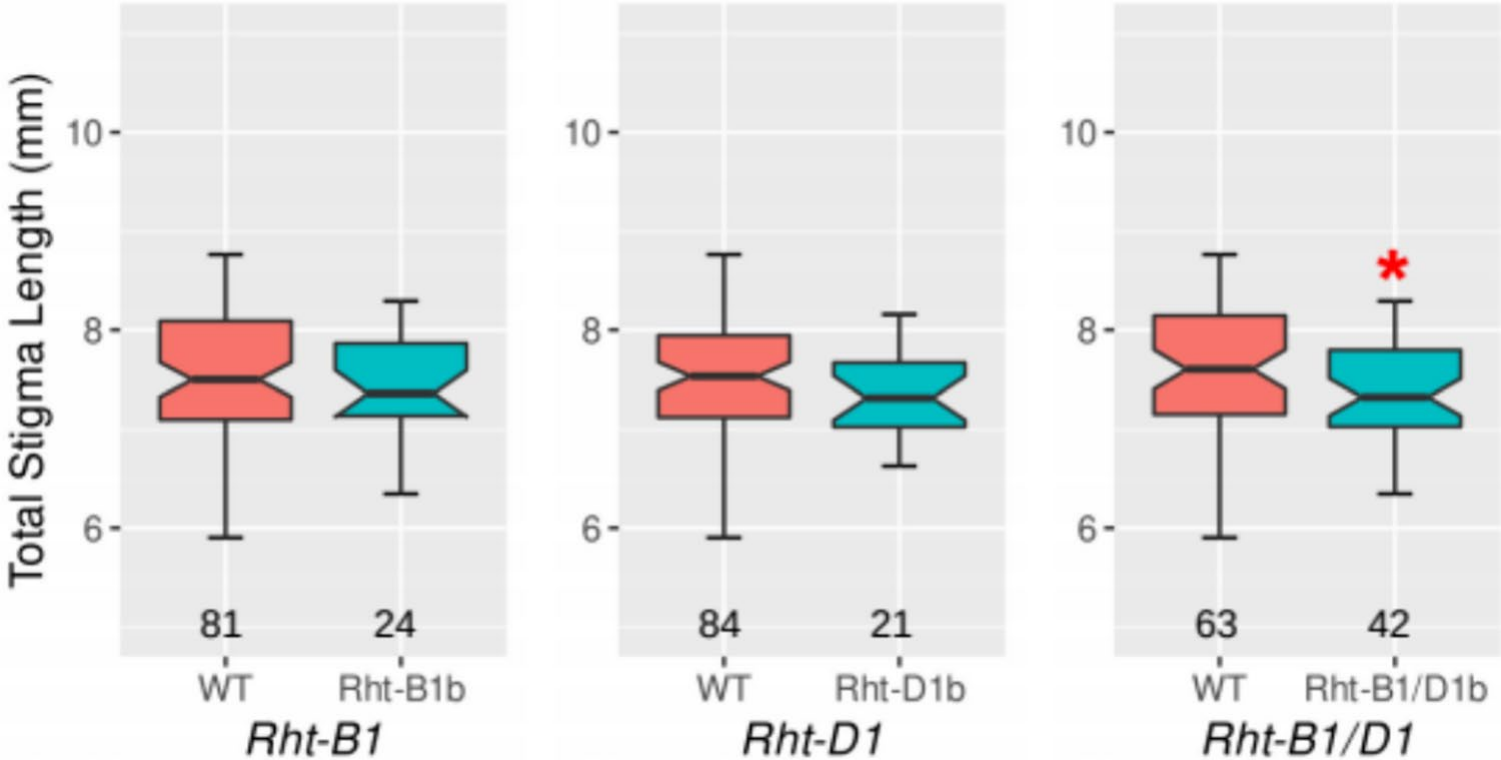
Association of *Rht-B1* and *Rht-D1* genotype with TSL in a diverse bread wheat panel of 116 lines. In the left panel, *Rht-B1* genotype on the X-axis is indicated as follows: WT; homozygous *Rht-B1a* allele (red bar), Rht-B1b; homozygous *Rht-B1b* semi-dwarf allele (blue bar). In the middle panel, *Rht-D1* genotype is indicated as follows: WT; homozygous *Rht-D1a* allele (red bar), Rht-D1b; homozygous *Rht-D1b* semi-dwarf allele (blue bar). *Rht* genotype in the right panel is shown as WT; homozygous for both *Rht-B1a* and *Rht-D1a* (red bar) and Rht-B1/D1b; homozygous for either *Rht-B1b* or *Rht-D1b* or both (blue bar). Number of plants for each genotype group is indicated at the bottom, and significant difference identified by Student’s *t*-test (*P* < 0.05) between groups is indicated by asterisk

Across the bread wheat panel values for average TSL ranged from 5.90 to 8.83 mm (Fig. 1; Table S1). Carpel hairs were not removed before imaging in this initial experiment and consequently standard deviation values were somewhat high. Nonetheless, we were able to confidently select lines at extremes as parents for mapping populations. As is evident in Table 1, testing of these parental lines in subsequent experiments confirmed their TSL phenotype. Given the diverse origins of our panel, we had anticipated a wide range in Days to Anthesis (DA) and found it to be 52–167 days. DA is known to impact many traits, often to a large extent, and can impede implementation of traits in breeding programs. Encouragingly, we found little correlation between DA and TSL (*R* = 0.0424), leading us to have greater confidence in the usefulness of our TSL evaluation method. PH was not measured because tillers were removed for stigma collection or were used for crossing, but the panel was genotyped for presence of *Rht-B1b* and/or *Rht-D1b* (Table S1). Evaluation of association between TSL and *Rht* allele type found no significant effect for either *Rht-B1b* or *Rht-D1b* (Fig. 2). A small but significant (*P* < 0.05) difference was observed when lines were grouped as wild type (*Rht-B1a* plus *Rht-D1a*) or containing either *Rht-B1b* or *Rht-D1b* or both, with TSL being lower in lines containing dwarfing alleles. Date of line origin, which was available for 76 of the lines (Supplementary Table S1), was examined for association with TSL. Arraying TSL values by release date revealed no obvious trend (*R* = − 0.0278), suggesting that TSL has not been under strong positive selection over the course of modern breeding. The remaining 40 lines were predominantly land-varieties and amongst these, we noted a TSL range and distribution pattern that was highly similar to that of the subset of lines with release year information.

**Table 1:** Trait values of parental lines

Line name	*Rht* status	Experiment	TSL (mm)	DA (days)	PH (cm)	PL (mm)	No. plants
Mexicali	*Rht1*	Panel-durum	7.12 ± 0.19	50	67.0	NA	1
		Expt-D1	7.80 ± 0.21	47.3 ± 0.6	69.7 ± 1.5	104.0 ± 7.2	3
		Expt-D2-sowing1	8.49 ± 0.17	44.5 ± 1.7	69.0 ± 1.4	159.7 ± 9.3	4
		Expt-D2-sowing2	8.38 ± 0.17	43.0 ± 0.0	65.5 ± 0.7	123.8 ± 0.7	2
Italo	*Rht14*	Panel-durum	9.77 ± 0.72	56	53.0	NA	1
		Expt-D1	10.58 ± 0.15	48.7 ± 1.2	51.7 ± 4.2	42.67 ± 2.3	3
		Expt-D2-sowing1	10.68 ± 0.24	48.3 ± 2.4	55.3 ± 2.6	62.9 ± 18.8	4
		Expt-D2-sowing2	10.95 ± 0.24	47.0 ± 1.4	52.5 ± 3.5	34.3 ± 17.9	2
Mocho	wt	Panel-bread	8.47 ± 0.12	103.00	163.0	NA	1
		Expt-MoGl-RIL	8.88 ± 0.29	118.7 ± 6.4	125.4 ± 5.4	NA	3
Gladius	*Rht2*	Expt-MoGl-RIL	7.34 ± 0.13	59.0 ± 1.0	62.0 ± 1.9	NA	2
Thori	wt	Panel-bread	8.10 ± 0.30	65	79.5	NA	1
		Expt-ThHy-F2	9.51 ± 0.25	51.7 ± 0.6	110.3 ± 7.0	NA	3
Hydra	*Rht1*	Panel-bread	7.11 ± 0.09	56	58.0	NA	1
		Expt-ThHy-F2	8.23 ± 0.33	55.0 ± 1.0	65.0 ± 3.6	NA	3

*NA* Data not available

The durum panel of 27 lines that included both elite cultivars and land-varieties had average TSL values that ranged from 7.12 to 9.77 mm (Fig. S1; Table S2). Even though the durum panel comprised considerably fewer lines than the bread wheat panel, the diversity in stigma presentation was pronounced. Selected examples are shown in Fig. 1. Notable lines include Kalka with its mid-length but highly ‘hairy’ stigma and the aforementioned carpel hairless Don Pedro. The small number of lines in the panel precluded meaningful analysis of the impact of DA on TSL, or association between *Rht-B1b* and TSL. However, as for the bread wheat panel, we noted no strong trend between TSL and DA, nor TSL and the presence of *Rht-B1b*. Again, PH was not measured because tillers were used for crossing.

Screening of the two panels provided sufficient information to select parents for durum and bread wheat population development. Attributes of these parents across growing seasons are shown in Table 1.

### TSL in near-isogenic sterile and fertile bread wheat backgrounds

For application to hybrid breeding, an important aspect to consider is whether TSL in a male sterile background differs significantly to that of its fertile counterpart. Casual observation of stigma appearance during carpel enlargement after pollination failure in both male sterile lines and emasculated florets indicated no obvious lengthening of the stigma. This led us to anticipate that TSL in fertile lines would accurately reflect TSL in male sterile derivatives. To test this hypothesis, we generated near-isogenic male sterile and fertile pairs by crossing bread wheats to *Ms3*/7*1IBWSN50 backcross lines heterozygous for the dominant *Ms3* male sterility gene and hence male sterile. As male parents for the crosses, we selected 9 bread wheats that ranged in TSL from low to high. We then measured TSL in sterile (*Ms3m*s*3*) and fertile (*ms3ms3*) F_1_ segregants for each cross. As shown in Fig. S2, we generally found highly similar mean TSL values for sterile and fertile segregants, suggesting that measurement in fertile lines should indeed reflect TSL of male sterile derivatives. As shown in Fig. S3, this experiment also indicated that TSL exhibits partial to full dominance. Partial dominance was also noted in a separate bread wheat experiment that comprised 10 sets of parental and F_1_ plants derived from crosses between lines of our screening panel, as well as in a durum wheat experiment involving three sets of parentals and F_1_ plants. In a hybrid breeding system that involved a female parent with high TSL and male parent with lower TSL, we anticipate that F_1_ plants would have a mid-range TSL.

### TSL in durum wheat Mexicali/Italo F_2_ population associates with *Rht14*

We selected Mexicali (low TSL) and Italo (high TSL) for population development not only because in our panel they represented the extremes of TSL phenotype, but also because Italo is a semi-dwarf line that does not contain *Rht-B1b*. Italo is a derivative of Castelpoziano and, like it, carries the *Rht14* mutation on chromosome 6A that results in reduced plant height but not the loss of GA-responsiveness. Such so called ‘alternative’ dwarfing genes are thought to offer multiple advantages, including improved seedling establishment and greater early vigour (Rebetzke et al. 2022). Choosing Italo provided us an opportunity to examine the effect on TSL of a GA-responsive dwarfing gene, whilst also examining the effect of the *Rht-B1b* allele derived from Mexicali.

As described in Methods, we examined Mexicali/Italo F_2_ plants in two separate spring season glasshouse experiments, Expt-D1 and Expt-D2 involving 90 and 180 F_2_ plants, respectively, with Expt-D2 sown across two dates. Because we identified an effect, *albeit* small, of sowing date on trait values our QTL model for Expt-D2 included sowing group. Overall, across the two seasons we found TSL values to be similar (Fig. S4), encouraging confidence in our trait measurement methodology and subsequent QTL identification.

As Expt-D1 progressed it became apparent that lines with larger TSL tended to have smaller peduncle length (PL), which suggested linkage between TSL and *Rht14* since reduced PL is a known effect of *Rht14* (Vikhe et al. 2019). Indeed, PH was highly correlated with PL in both experiments (Fig. S5B). Correlation analysis also confirmed the strong negative correlation between TSL and PL and between TSL and PH (Fig. S5C, D). These observations led us to employ a targeted mapping strategy towards examining the association of TSL with *Rht14*. Our marker set (Table S4) included cs214 and cs412 which closely flank *Rht14*, and a further 26 markers that cover most of chromosome 6A, spanning approximately 600 Mb of the Svevo.v1 reference sequence. Because durum wheat is an allo-tetraploid, we also included 7 markers that locate to the homeologous *Rht14* region on chromosome 6B. Additionally we included a marker for the Mexicali-derived *Rht-B1b* allele on chromosome 4B. We also employed BLAST searches using rice SYL3-k coding sequence as bait against Svevo durum wheat (Maccaferri et al. 2019) and Chinese Spring bread wheat (International Wheat Genome Sequencing Consortium 2018) to identify potential orthologues to include as additional markers, but found none. We did not include markers for major flowering phenology genes because all F_2_ plants were spring type and flowering occurred within 10 days in Expt-D1 and 10 and 12 days in the two Expt-D2 sowing groups. Results from Expt-D1 informed selection of a marker subset for genotyping in Expt-D2; 12 robust 6A markers that span the *Rht14* region, four chromosome 6B markers and the marker for *Rht-B1b*.

**Fig. 3:**
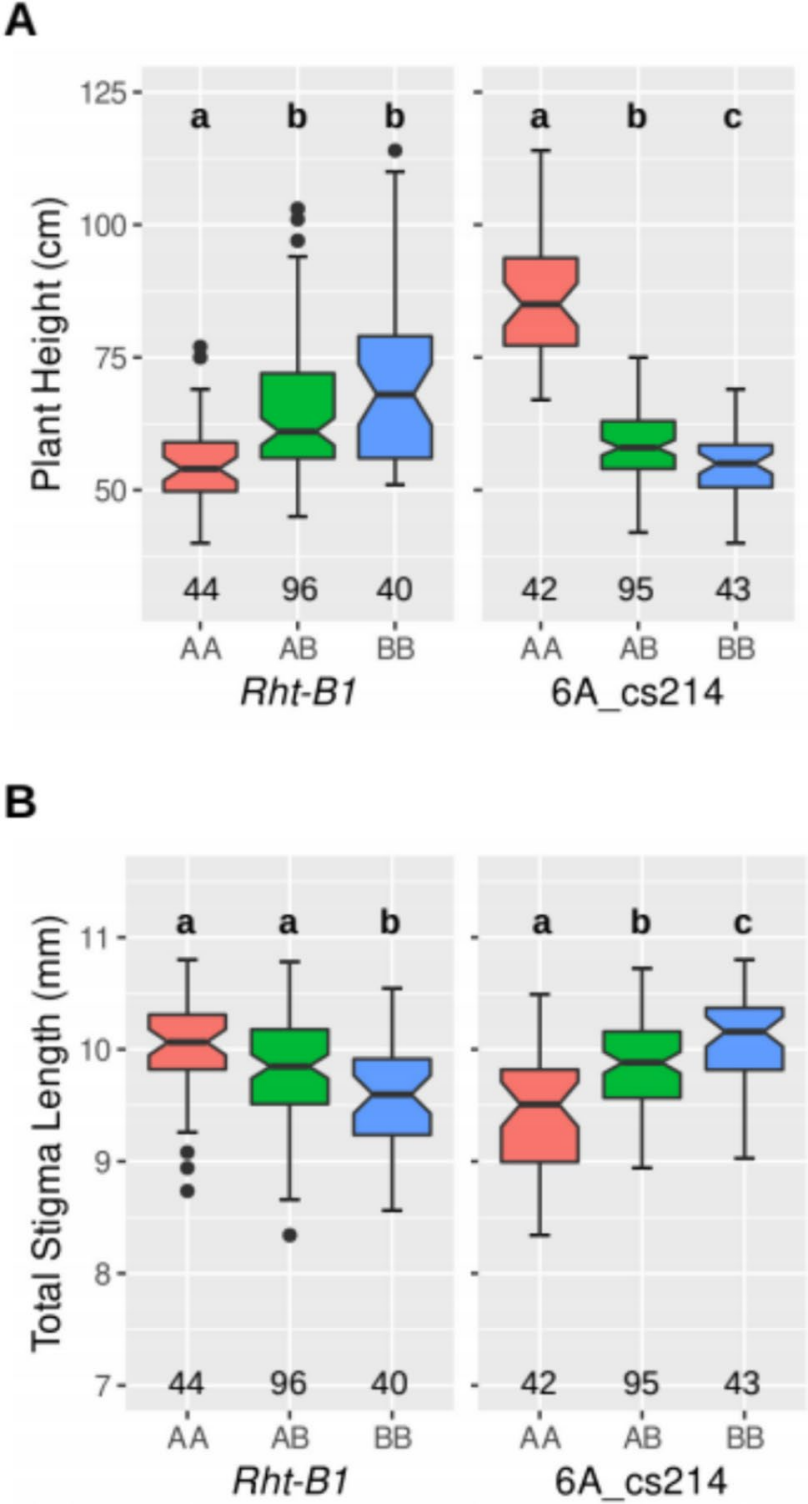
Association of PH (**A**) and TSL (**B**) with QTL marker genotype in Mexicali/Italo F_2_ Expt-D2. Homozygous (AA; Mexicali-derived, BB; Italo-derived) or heterozygous (AB) genotypes are indicated on the X-axis for markers *Rht-B1* (left panel) and cs214 (right panel). Number of plants in each genotype group is indicated at the bottom, and groups with different index letters are significantly different by ANOVA with Tukey’s test at *P* < 0.05

Results of linkage analyses are summarised in Table 2, with specific allele effects on TSL and PH shown in Fig. 3. As expected for this population, in both Expt-D1 and Expt-D2, the *Rht14* allele of Italo and the *Rht-B1b* allele of Mexicali were associated with reduced PH and reduced PL, with the *Rht14* allele having greater effect on both traits. In Expt-D1 and Expt-D2, respectively, reduction in PH due to the *Rht14* locus was 29.4 and 31.5 cm compared to 19.0 and 15.4 cm for *Rht-B1b* locus*,* whilst PL reduction was 124.8 and 118.2 mm for the *Rht14* locus and 57.2 and 35.0 mm for *Rht-B1b* locus (Table 2). The strong effect of the *Rht14* allele on PL is consistent with findings in previous *Rht14* studies (Vikhe et al. 2019). As mentioned above, the range in DA was small in both experiments, but the cs214 Italo allele was found to be associated with a slight increase in DA in both experiments (Table 2).

**Table 2:** QTL detected in the Mexicali/Italo F_2_ population

QTL name	Trait^a^	Experiment	Chr	Marker^b^	LOD^c^	% Pv^d^	AA mean^e^	BB mean
Total stigma length (mm)								
*Qtsl.ua-6A.Expt1.1*	TSL	Expt-D1	6A	cs214	5.87	25.2	8.78	9.65
*Qtsl.ua-6A.Expt2.1*	TSL	Expt-D2	6A	cs214	9.29	19.2	9.43	10.08
*Qtsl.ua-4B.Expt2.1*	TSL	Expt-D2	4B	Rht1	3.53	6.8	10.01	9.58
Days to Anthesis (days)								
*Qda.ua-6A.Expt1.1*	DA	Expt-D1	6A	cs214	2.96	14.0	49.1	51.0
*Qda.ua-6A.Expt2.1*	DA	Expt-D2	6A	cs214	2.98	7.1	46.8	48.1
Plant Height (cm)								
*Qph.ua-4B.Expt1.1*	PH	Expt-D1	4B	Rht1	19.00	25.7	59.6	78.6
*Qph.ua-6A.Expt1.1*	PH	Expt-D1	6A	cs214	31.57	63.0	86.1	56.7
*Qph.ua-4B.Expt2.1*	PH	Expt-D2	4B	Rht1	20.91	9.7	55.3	70.7
*Qph.ua-6A.Expt2.1*	PH	Expt-D2	6A	cs214	68.88	66.2	86.3	54.8
Peduncle Length (mm)								
*Qpl.ua-4B.Expt1.1*	PL	Expt-D1	4B	Rht1	23.06	19.2	71.0	128.2
*Qpl.ua-6A.Expt1.1*	PL	Expt-D1	6A	cs214	44.93	76.5	175.0	50.2
*Qpl.ua-4B.Expt2.1*	PL	Expt-D2	4B	Rht1	5.58	3.2	86.2	121.2
*Qpl.ua-6A.Expt2.1*	PL	Expt-D2	6A	cs214	53.66	60.6	191.1	72.9

^a^Traits used for QTL analysis. *DA* days to anthesis, *PL* peduncle length, *PH* plant height, *TSL* total stigma length

^b^Marker closest to QTL peak

^c^LOD score for QTL peak

^d^% Phenotypic variation explained by the QTL

^e^Mean trait value of lines homozygous for Mexicali (AA) or Italo (BB) alleles

For TSL, we found that cs214, the *Rht14*-linked marker on 6A, explained 25.2% and 19.2% of phenotypic variation in Expt-D1 and Expt-D2, respectively, with the Italo-derived locus contributing on average higher TSL values of 0.87 and 0.65 mm. Corresponding LOD values for the associations were 5.87 and 9.29, respectively. The higher LOD value in Expt-D2 is likely attributable to the greater number of individuals in that experiment (*N* = 180 in Expt-D2 compared with *N* = 90 in Expt-D1). In Expt-D2 only, the *Rht-B1b* dwarfing allele of Mexicali was associated with a minor positive effect on TSL (LOD = 3.53), explaining 6.8% of phenotypic variation. Therefore, in our targeted mapping analysis, the *Rht14* locus exhibited a significantly stronger association with TSL compared to the *Rht-B1* locus. Intriguingly, the dwarfing allele of the *Rht14* locus derived from Italo is linked to an extended stigma length.

### Multiple genetic loci contribute to TSL in bread wheat population Mocho/Gladius RIL

The greater complexity of the bread wheat genome led us to expect difficulty in resolving genetic effects for TSL in F_2_ populations, we therefore undertook analysis in a RIL population developed from a cross between Mocho de Espiga Branca and Gladius, lines that contrast in TSL (Fig. 1; Table 1). For simplicity, here we abbreviate Mocho de Espiga Branca as ‘Mocho’. As shown in Table 1, Gladius carries the *Rht-D1b* allele for reduced height and is semi-dwarf in stature with low TSL, whilst Mocho is wild type at both *Rht-B1* and *Rht-D1* and is very tall with high TSL. Mocho, although classified as spring type, was much later to anthesis than Gladius under our growth conditions. In this experiment we were somewhat hampered by intermittent occurrences of pistilloidy which reduced the number of usable stigma samples. In total, we measured TSL for 139 RILs. The population varied greatly for both DA (49–163 days) and PH (53.1 to 160.1 cm). The observed range in average TSL was 6.167–10.244 mm. Frequency distributions for these three traits are provided in Fig. S6.

Our trait correlation analysis found a moderately significant negative correlation of DA with TSL, suggesting an effect of flowering time on TSL. The analysis found no significant correlation between PH and TSL (Fig. S7), which is different from the result found for the durum wheat Mexicali/Italo F_2_ population (Fig. S5) where the *Rht14*-associated locus had a strong effect on both traits.

We generated a genetic map (Fig. S8) from 2774 genome-by-sequencing (GBS) markers (Table S3) as well as KASP™ markers for major phenology genes, including *Ppd-B1*on chromosome 2B and *Rht-D1* on chromosome 4D. Marker density was considered to be adequate for most A- and B-genome chromosomes, but as is typical for bread wheats, there is a paucity of D-genome polymorphism between our parents and consequently marker coverage across this genome is sporadic. QTL analysis for the traits TSL, DA and PH was performed and detected QTL are summarised in Table 3. For TSL, three weak to moderate associations were found. A marker in the region of the *Ppd-B1* locus on 2B showed the highest effect at LOD = 6.60, explaining 16.10% of phenotypic variation (% Pv). *Ppd-B1* was also strongly associated with DA (LOD = 78.12, % Pv = 63.77), consistent with the observed correlation between DA and TSL (Fig. S7). On average, the Mocho allele at *Ppd-B1* increased DA by 45.9 days and reduced TSL by 0.39 mm. Two additional minor QTLs for TSL were detected, one distally on chromosome arm 1AS (LOD = 4.70, % Pv = 11.08) and the other on chromosome arm 2AS (LOD = 4.12, % Pv = 9.62). The map had sufficient marker coverage across the region on chromosome 6A that was associated with TSL in the durum population, but no QTL was detected at that location. As expected, PH was strongly associated with *Rht-D1* (LOD = 61.48, % Pv = 53.96). The *Rht-D1b* allele from Gladius reduced height by an average of 28.3 cm. Importantly, and echoing our observation in the broader wheat panel, *Rht-D1b* showed no significant association with TSL. Allele effects for key QTL are demonstrated by box plots in Fig. S9. In summary, multiple weak to moderate QTLs contribute to TSL in the Mocho/Gladius population with no significant effect of the dwarfing allele *Rht-D1b*.

**Table 3:** QTL detected in the Mocho/Gladius RIL population

QTL name	Trait^a^	Chr	Position (cM)^b^	Marker^c^	LOD^d^	% Pv^e^	AA mean^f^	BB mean
Total stigma length (mm)								
*Qtsl.ua-1A.1.mch*	TSL	1A	34.0	MG_0040	4.70	11.08	8.35	8.02
*Qtsl.ua-2A.1.mch*	TSL	2A	50.9	MG_0549	4.12	9.62	8.41	7.90
*Qtsl.ua-2B.1.gld*	TSL	2B	71.0	MG_0666	6.60	16.10	7.93	8.32
Days to anthesis (days)								
*Qda.ua-2B.1.gld*	DA	2B	66.0	Ppd-B1	78.12	63.77	114.0	68.1
Plant height (cm)								
*Qph.ua-2B.1.gld*	PH	2B	66.0	Ppd-B1	14.11	8.36	90.0	83.4
*Qph.ua-4D.1.gld*	PH	4D	0.0	Rht-D1	61.48	53.96	100.0	71.7
*Qph.ua-5A.1.gld*	PH	5A	128.1	MG_1643	4.52	2.48	90.5	83.2

^a^Traits used for QTL analysis. *DA* days to anthesis, *PH* plant height, *TSL* total stigma length

^b^Peak QTL position in the genetic linkage map

^c^Marker closest to the QTL peak

^d^LOD score for QTL peak

^e^% Phenotypic variation explained by the QTL

^f^Mean trait value of lines homozygous for Mocho (AA) or Gladius (BB) alleles

### *Rht-B1b* has weak effect on TSL in bread wheat Thori/Hydra F_2_ population

The dwarfing allele *Rht-B1b* did not show an effect on TSL in our bread wheat diversity panel (Fig. 2), whilst analysis of the durum wheat Mexicali/Italo F2 population revealed a weak but significant effect of *Rht-B1b* on TSL in Expt-D2 ( Fig. 3B; Table 2). To examine the effect of *Rht-B1b* on TSL in a bread wheat biparental population, we used F_2_ plants from the cross Thori/Hydra. As shown in Table 1, Hydra carries the *Rht-B1b* allele for reduced height and is semi-dwarf in stature, whilst Thori is wild type at both *Rht-B1* and *Rht-D1* and is moderately tall. Although no genetic map was available for QTL analysis, the population was useful for examining the specific effect of *Rht-B1* on TSL. Genotypic and phenotypic data for 82 individuals revealed a small but significant (*P* < 0.05) effect, with the dwarfing *Rht-B1b* allele reducing TSL by an average of 0.462 mm (Fig. S10). Correlation analysis between TSL and PH returned a value of *R* = 0.36, consistent with *Rht-B1b* being the primary height-reducing agent in this population. Whilst the observation of *Rht-B1b* reducing TSL in this population is opposite to the effect found in the Mexicali/Italo study, where the *Rht-B1b* dwarfing allele was associated with a slightly higher TSL in Expt-D2 (Fig. 3), both effects were minor. DA varied by 54 days (39–92) but most lines had flowered by day 66, with only five lines flowering beyond that day. No correlation between TSL and DA was observed. Unfortunately, no markers within our durum chromosome 6A subset of TSL-linked markers were polymorphic between Thori and Hydra, and therefore, association of that region with TSL was not tested in this study. In summary, the bread wheat Thori/Hydra F_2_ population analysis indicates that *Rht-B1* exerts only minor effect on TSL, in line with results from the bread wheat diversity panel analysis.

### Transfer of high-TSL locus from durum cv. Italo to bread wheat

To test whether the high TSL 6A locus of Italo is transferrable to bread wheat we initiated marker-assisted backcrossing into a bread wheat line. A *Ms2* backcross line, *Ms2*/6*SUN276A, was chosen as the bread wheat parent to facilitate examination of the performance of the Italo-derived locus in male sterile plants. The *Ms2* mutation behaves dominantly and causes male sterility when heterozygous, reducing the number of generations required to obtain male sterile plants for analysis. After the initial cross was made, to rapidly generate backcross lines with a full 2*n* = 42 chromosome complement, we used partially fertile *ms2*/*ms2* F_1_ lines as pollen donors. The rationale of using plants that were 2*n* = 14 pairs + 7 univalents as male parents for the first backcross, despite their low fertility and the extra effort required to generate grain, was to take advantage of male gamete selection pressure. Following the same crossing strategy, material was advanced to BC_2_F_1_ plants and examined for both chromosome composition and TSL. Amongst the 16 plants examined, seven contained a full hexaploid content of 2*n* = 42 chromosomes. TSL and PH for these seven lines are shown in Fig. S11. The transferred locus, where present, was in a heterozygous state as these lines were direct products of crosses. Whilst the number of lines was too small for robust conclusion, BC_2_F_1_ plants which were heterozygous for the *Rht14* allele showed significantly (*P* < 0.05) higher TSL (Fig. S11). In addition, visual examination of stigma exsertion in the one line that was sterile and contained a single dose of *Rht14* was highly suggestive of improvement over that of sterile hexaploid *Ms2*/6*SUN276A parent plants. These encouraging observations warrant further investigation.

## Discussion

In this study, we developed a measurement method for wheat stigma length, characterised the genetic diversity of TSL in both bread and durum wheat, and identified genetic loci associated with this trait. A feature noted throughout our study was genotypic reproducibility of durum and bread wheat TSL differences across seasons and years. This reproducibility is likely a consequence of our standardised strict sampling and imaging methods. Jiang et al. (2021) also identified methodology standardisation of samples collected at anthesis as critical for generating consistent and reliable data for genetic analysis. Further advantage was gained by our use of glasshouse-grown plants which allowed careful developmental stage determination, versus the use of field-grown plants by Jiang et al. (2021). However, regardless of improvement in accuracy being gained by standardised sampling, the need for manual image analysis in calculating TSL remains laborious. A recent study on the application of machine learning towards automated image analysis potentially provides a framework for more efficient measurement of size and structure related stigma attributes (Millan-Blanquez et al. 2022). Such approaches should facilitate effective large-scale studies, although the effort required for monitoring of developmental stage, sample collection, stigma excision and imaging remain crucial and somewhat labour-intensive.

The genotypic variation for stigma presentation revealed by this study provides the basis for future genetic analyses, particularly for those female reproductive traits aimed at lowering the cost of hybrid grain production. Major phenology loci are well-known to have confounding effects on a multitude of traits, whether that be in conventional or hybrid systems. In this context, we aimed to determine whether TSL was affected by flowering time. For both the bread wheat panel and the Thori/Hydra F_2_ population, no association between DA and TSL was found (Sup Fig. S10). Contrastingly, in the Mocho/Gladius RIL population, we observed a significant association (LOD = 6.6, % Pv = 16.10) between *Ppd-B1* and TSL, with early flowering associating with longer stigma (Sup Fig. S9; Table 3). As with other traits that are influenced by *Ppd-B1*, further understanding of how specific *Ppd-B1* alleles affect TSL will be necessary before implementing selection for high TSL in a breeding context. In our durum population, there was insufficient variation in DA to fully assess the impact of DA on TSL, but the limited observations indicated no obvious trend.

Along with genes controlling flowering time, the semi-dwarfing mutant alleles *Rht-B1b* and *Rht-D1b* are particularly important in breeding and are similarly known to impact numerous traits, including reducing anther length and extrusion. The structure of our bread wheat populations facilitated assessment of these mutant alleles separately, whilst our durum population allowed assessment of *Rht-B1b*, *albeit* in conjunction with segregation for *Rht14*. Considering *Rht-B1b*, we found that in the bread wheat Thori/Hydra population this allele was associated with a slight decrease in TSL, whereas in the durum wheat Mexicali/Italo population, this allele was associated with a slight increase in TSL in just one of the two experiments (Expt-D2). We surmise that the dwarfing gene *Rht-B1b* may have a minor effect on TSL depending on wheat genotype. In the case of *Rht-D1b*, which was specifically assessed in the bread wheat Mocho/Gladius RIL population, no association with TSL was identified. Encouragingly, no association with TSL was found across the bread wheat panel for either *Rht-B1b* or *Rht-D1b* although if lines were pooled as either wild type or containing one or more of *Rht-B1b* and *Rht-D1b* a slight reducing effect on TSL was evident (Fig. 2). In summary, of the multiple experiments conducted here none identified a strong effect of either *Rht-B1b* or *Rht-D1b* on TSL.

This study is the first to report that increased TSL associates with the *Rht14* allele of Italo providing the opportunity to simultaneously select for reduced plant stature and improved stigma exsertion, two components important for breeding a female parental ideotype for efficient hybrid grain production (Whitford et al. 2013). Positional cloning studies of both *Rht14* and the allelic *Rht18* (Ford et al. 2018; Haque et al. 2011; Vikhe et al. 2019) propose involvement of *GA2oxA9* (TRIT6Av1G140910), a GA 2-oxidase, in the dwarfing phenotype. *Rht14* locates to a centromeric region of chromosome 6A that has reduced recombination, with Ford et al. (2018) reporting a genetic to physical ratio of 1 cM/230 Mb in an Icaro/Langdon F_2_ population segregating for the allelic *Rht18*. This means that either studies using very large populations or alternative approaches are needed to test whether *Rht14* can be uncoupled from the TSL QTL identified here. Whilst our attempt to transfer the long stigma trait from Italo to bread wheat appeared to be encouraging, it is currently unknown how effective *Rht14* may be in reducing bread wheat height in contemporary cultivars. Backcrossing of *Rht18* from durum cultivar Icaro into the 1969-released tall bread wheat cultivar Halberd reduced plant height by 24% (Rebetzke et al. 2022), however, as revealed in Würschum et al. (2017) and Tian et al. (2022), contemporary bread wheat cultivars frequently contain the dwarfing gene *Rht24* in addition to either *Rht-B1b* or *Rht-D1b*, with the presence of *Rht24* increasing dwarfing beyond that due to *Rht-B1b* or *Rht-D1b*. It is currently not clear whether *Rht14* from Italo would be more effective than the *Rht24b* allele in decreasing height in bread wheat but examining whether TSL is associated with *Rht24* is worthy of future investigation. The Mexicali/Italo population proved to be amenable for investigating genetic control of stigma length and development of a full genetic map for the population could reveal additional useful QTL.

In the Mocho/Gladius RIL population QTL analysis revealed no additional major effect loci for TSL other than *Ppd-B1*. This can potentially be attributed to either the extra complexity of hexaploid genome relative to tetraploid, lack of full genome marker coverage or lack of sufficient population size. The high incidence of pistilloidy in the population reduced the total number of individuals for which TSL could be reliably measured, decreasing QTL detection power. There are, however, future opportunities for developing other bread wheat mapping populations based on TSL ranking of the 116 lines in our bread wheat panel.

Additional research is required to confirm that longer stigma positively contribute to improved hybrid wheat grain set, although such a link has recently been reported in rice (Dang et al. 2022). For this purpose, contrasting allelic *Ms2*/*Rht14* isolines could be further developed and field tested for grain set following cross-pollination. The *Ms3* isolines developed in this study could also be used towards this goal. Or chemical hybridising agents such as Croisor 100® or Genesis® could be employed for isoline studies not involving genetic male steriles.

Beyond studies of stigma length, the existence of significant pistil diversity in both hexaploid and tetraploid wheats poses intriguing questions, such as what might be the role of carpel hairs and why are they are so diminished in some durum lines? And has there been any positive selection for reduced stigma ‘hairiness’ and stigma protrusion in geographical regions that experience a high incidence of heat and drought stress, given that these structures are particularly susceptible to low humidity induced atrophy? Stigma receptiveness and receptivity duration were not studied here but are clearly highly important traits. Our success in achieving reproducible TSL values by employing strict methodology may encourage studies on stigma receptivity. What is certain is that understanding and exploiting stigma diversity will provide opportunities for gain in the quest for cost-effective hybrid grain production.

### Supplementary Information

Below is the link to the electronic supplementary material.

Supplementary file1 (PDF 849 kb)

Supplementary file2 (PDF 107 kb)

Supplementary file3 (XLSX 425 kb)

## References

[CR1] Akel W, Rapp M, Thorwarth P, Wurschum T, Longin CFH (2019). Hybrid durum wheat: heterosis of grain yield and quality traits and genetic architecture of anther extrusion. Theor Appl Genet.

[CR2] Borjigin C, Schilling RK, Jewell N, Brien C, Sanchez-Ferrero JC, Eckermann PJ, Watson-Haigh NS, Berger B, Pearson AS, Roy SJ (2021). Identifying the genetic control of salinity tolerance in the bread wheat landrace Mocho de Espiga Branca. Funct Plant Biol.

[CR3] Broman KW, Wu H, Sen S, Churchill GA (2003). R/qtl: QTL mapping in experimental crosses. Bioinformatics.

[CR4] Dang X, Zhang Y, Li Y, Chen S, Liu E, Fang B, Liu Q, She D, Dong Z, Fan Z, Li D, Wang H, Zhu S, Hu X, Li Y, Jiang J, Hong D (2022). *SYL3-k* increases style length and yield of F1 seeds via enhancement of endogenous GA4 content in *Oryza sativa* L. pistils. Theor Appl Genet.

[CR5] De Vries AP (1971). Flowering biology of wheat, particularly in view of hybrid seed production—a review. Euphytica.

[CR6] Duan S, Cui C, Chen L, Yang Z, Hu Y-G (2022). Fine mapping and candidate gene analysis of dwarf gene Rht14 in durum wheat (Triticum durum). Funct Integr Genom.

[CR7] El Hanafi S, Cherkaoui S, Kehel Z, Al-Abdallat A, Tadesse W (2021). Genome-wide association and prediction of male and female floral hybrid potential raits in elite spring bread wheat genotypes. Plants.

[CR8] El-Namaky R (2018). The genetic variability of floral and agronomic characteristics of newly-bred cytoplasmic male sterile rice. Agriculture.

[CR9] Ford BA, Foo E, Sharwood R, Karafiatova M, Vrána J, MacMillan C, Nichols DS, Steuernagel B, Uauy C, Doležel J, Chandler PM, Spielmeyer W (2018). *Rht18* semidwarfism in wheat Is due to increased GA 2-oxidaseA9 expression and reduced GA content. Plant Physiol.

[CR10] Gupta R, Sutradhar H, Chakrabarty SK, Ansari MW, Singh Y (2015). Stigmatic receptivity determines the seed set in Indian mustard, rice and wheat crops. Commun Integr Biol.

[CR11] Haque MA, Martinek P, Watanabe N, Kuboyama T (2011). Genetic mapping of gibberellic acid-sensitive genes for semi-dwarfism in durum wheat. Cereal Res Commun.

[CR12] He X, Singh PK, Dreisigacker S, Singh S, Lillemo M, Duveiller E (2016). Dwarfing genes *Rht-B1b* and *Rht-D1b* are associated with both Type I FHB susceptibility and low anther extrusion in two bread wheat populations. PLoS ONE.

[CR13] Imrie BC (1966). Stigma receptivity in cytoplasmic male sterile wheat. Aus J Exp Agric Anim Husb.

[CR14] International Wheat Genome Sequencing Consortium (2018). Shifting the limits in wheat research and breeding using a fully annotated reference genome. Science.

[CR15] Jiang J, Xu L, Xiao M, Hu C, Zhang Y, Wang D, Dang X (2021). Genetic analysis and QTLs identification of stigma traits in japonica rice (*Oriza sativa* L.). Euphytica.

[CR16] Lu Q, Lillemo M, Skinnes H, He X, Shi J, Ji F, Dong Y, Bjornstad A (2013). Anther extrusion and plant height are associated with Type I resistance to *Fusarium* head blight in bread wheat line 'Shanghai-3/Catbird'. Theor Appl Genet.

[CR17] Maccaferri M (2019). Durum wheat genome highlights past domestication signatures and future improvement targets. Nat Genet.

[CR18] Millan-Blanquez M, Hartley M, Bird N, Manes Y, Uauy C, Boden SA (2022). A scalable phenotyping approach for female floral organ development and senescence in the absence of pollination in wheat. Development.

[CR19] Muqaddasi QH, Lohwasser U, Nagel M, Borner A, Pillen K, Roder MS (2016). Genome-wide association mapping of anther extrusion in hexaploid spring wheat. PLoS ONE.

[CR20] Muqaddasi QH, Brassac J, Borner A, Pillen K, Roder MS (2017). Genetic architecture of anther extrusion in spring and winter wheat. Front Plant Sci.

[CR21] Muqaddasi QH, Jayakodi M, Borner A, Roder MS (2019). Identification of consistent QTL with large effect on anther extrusion in doubled haploid populations developed from spring wheat accessions in German Federal *ex situ* Genebank. Theor Appl Genet.

[CR22] Okada T, Jayasinghe JEARM, Nansamba M, Baes M, Warner P, Kouidri A, Correia D, Nguyen V, Whitford R, Baumann U (2017). Unfertilized ovary pushes wheat flower open for cross-pollination. J Exp Bot.

[CR23] Okada T, Jayasinghe J, Eckermann P, Watson-Haigh NS, Warner P, Hendrikse Y, Baes M, Tucker EJ, Laga H, Kato K, Albertsen M, Wolters P, Fleury D, Baumann U, Whitford R (2019). Effects of *Rht-B1* and *Ppd-D1* loci on pollinator traits in wheat. Theor Appl Genet.

[CR24] Okada T, Jayasinghe J, Eckermann P, Watson-Haigh NS, Warner P, Williams ME, Albertsen MC, Baumann U, Whitford R (2021). Genetic factors associated with favourable pollinator traits in the wheat cultivar Piko. Funct Plant Biol.

[CR25] Pallotta MA, Graham RD, Langridge P, Sparrow DH, Barker SJ (2000). RFLP mapping of manganese efficiency in barley. Theor Appl Genet.

[CR26] Pallotta MA, Warner P, Fox RL, Kuchel H, Jefferies SJ, Langridge P, Pogna NE (2003). Marker assisted wheat breeding in the southern region of Australia. Proc 10th Int wheat genet symp istituto sperimentale per la Cerealicoltura.

[CR27] Pallotta M, Schnurbusch T, Hayes J, Hay A, Baumann U, Paull J (2014). Molecular basis of adaptation to high soil boron in wheat landraces and elite cultivars. Nature.

[CR28] Prahalada GD, Marathi B, Vinarao R, Kim S-R, Diocton R, Ramos J, Jena KK (2021). QTL mapping of a novel genomic region associated with high out-crossing rate derived from *Oryza longistaminata* and development of new CMS lines in rice *O. Sativa* L. Rice.

[CR29] Rasheed A, Wen W, Gao F, Zhai S, Jin H, Liu J, Guo Q, Zhang Y, Dreisigacker S, Xia X, He Z (2016). Development and validation of KASP assays for genes underpinning key economic traits in bread wheat. Theor Appl Genet.

[CR30] Rebetzke GJ, Rattey AR, Bovill WD, Richards RA, Brooks BJ, Ellis M (2022). Agronomic assessment of the durum *Rht18* dwarfing gene in bread wheat. Crop Pasture Sci.

[CR31] RStudio Team (2020) RStudio: integrated development for R. RStudio. PBC. http://www.rstudio.com/. Accessed 14 Apr 2024

[CR32] Sade B, Ibrahim AMH, Subramanian N, Rudd J, Liu S, Opena G, Baenziger S (2022). Assessment of floral characteristics for hybrid wheat (*Triticum aestivum* L.) production in Texas. Agrosyst Geosci Environ.

[CR33] Schindelin J, Arganda-Carreras I, Frise E, Kaynig V, Longair M, Pietzsch T, Preibisch S, Rueden C, Saalfeld S, Schmid B (2012). Fiji: an open-source platform for biological-image analysis. Nat Methods.

[CR34] Shang L, Song J, Yu H, Wang X, Yu C, Wang Y, Li F, Lu Y, Wang T, Ouyang B, Zhang J, Larkin RM, Ye Z, Zhang Y (2021). A mutation in a C2H2-type zinc finger transcription factor contributed to the transition toward self-pollination in cultivated tomato. Plant Cell.

[CR35] Singh SK, Arun B, Joshi AK (2007). Comparative evaluation of exotic and adapted germplasm of spring wheat for floral characteristics in the Indo-Gangetic Plains of northern India. Plant Breed.

[CR36] Skinnes H, Semagn K, Tarkegne Y, Maroy AG, Bjornstad A (2010). The inheritance of anther extrusion in hexaploid wheat and its relationship to *Fusarium* head blight resistance and deoxynivalenol content. Plant Breed.

[CR37] Takano-Kai N, Doi K, Yoshimura A (2011). GS3 participates in stigma exsertion as well as seed length in rice. Breed Sci.

[CR38] Tian X, Xia X, Xu D, Liu Y, Xie L, Hassan MA, Song J, Li F, Wang D, Zhang Y, Hao Y, Li G, Chu C, He Z, Cao S (2022). *Rht24b*, an ancient variation of TaGA2ox-A9, reduces plant height without yield penalty in wheat. New Phytol.

[CR39] Vikhe P, Venkatesan S, Chavan A, Tamhankar S, Patil R (2019). Mapping of dwarfing gene *Rht14* in durum wheat and its effect on seedling vigor, internode length and plant height. Crop J.

[CR40] Whitford R, Fleury D, Reif JC, Garcia M, Okada T, Korzun V, Langridge P (2013). Hybrid breeding in wheat: technologies to improve hybrid wheat seed production. J Exp Bot.

[CR41] Würschum T, Langer SM, Longin CFH, Tucker MR, Leiser WL (2017). A modern Green Revolution gene for reduced height in wheat. Plant J.

[CR42] Wurschum T, Liu G, Boeven PHG, Longin CFH, Mirdita V, Kazman E, Zhao Y, Reif JC (2018). Exploiting the *Rht* portfolio for hybrid wheat breeding. Theor Appl Genet.

[CR43] Zeven AC (1968). Cross pollination and sources of restorer genes in wheat and a semi-hybrid wheat variety. Euphytica.

[CR44] Zhou H (2017). Genome-wide association analyses reveal the genetic basis of stigma exsertion in rice. Mol Plant.

