# Supplementary material for: Diversity in bread and durum wheat stigma morphology and linkage of increased stigma length to dwarfing gene Rht14
Source: Theor Appl Genet. 2024 Jun 14;137(7):160. doi: 10.1007/s00122-024-04663-4 (PMC11178622; doi:10.1007/s00122-024-04663-4)
Supplement: Supplementary file 1 — Supplementary file1 (PDF 849 kb) [file 122_2024_4663_MOESM1_ESM.pdf]

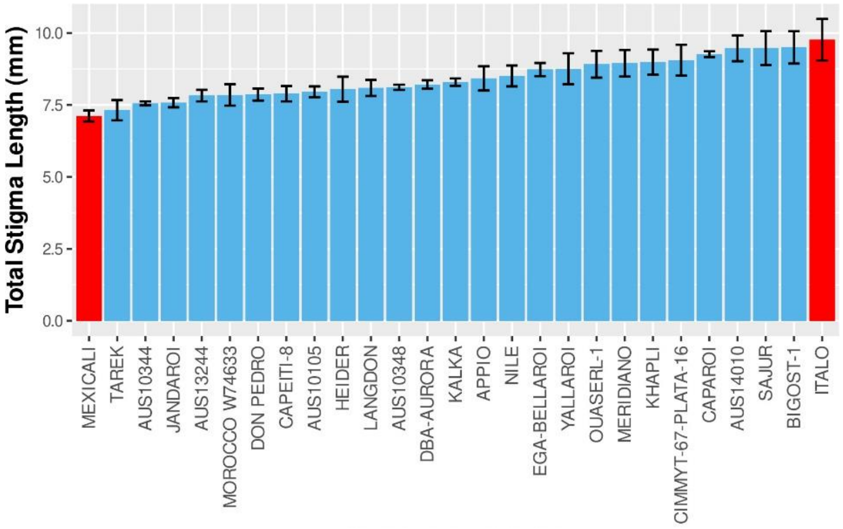

Figure S1. Distribution of TSL in a diverse panel of 27 durum wheats grown in a glasshouse. The Y-axis indicates average TSL, and error bars represent standard deviation. Along the X-axis, lines are ordered from lesser (left) to higher (right) TSL. Red bars indicate mapping population parents Mexicali and Italo.

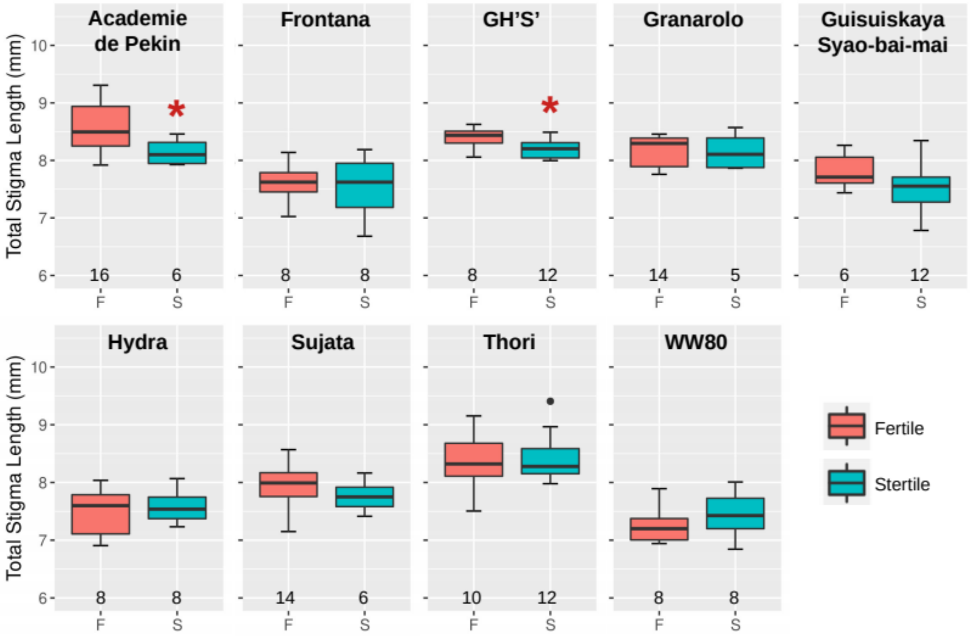

Figure S2. Effect of male sterility on TSL in near-isogenic F_1 plants. Nine bread wheat cultivars were crossed with *Ms3ms3* male sterile derivatives of *Ms3*/7*1IBWSN50 and F_1 progeny were investigated for TSL and fertility. Number of pistils used for TSL measurement from male fertile (F) or sterile (S) plants are indicated at the bottom of each panel. Significant differences identified by Student's *t*-test $p < 0.05$ between paired groups are indicated by asterisk.

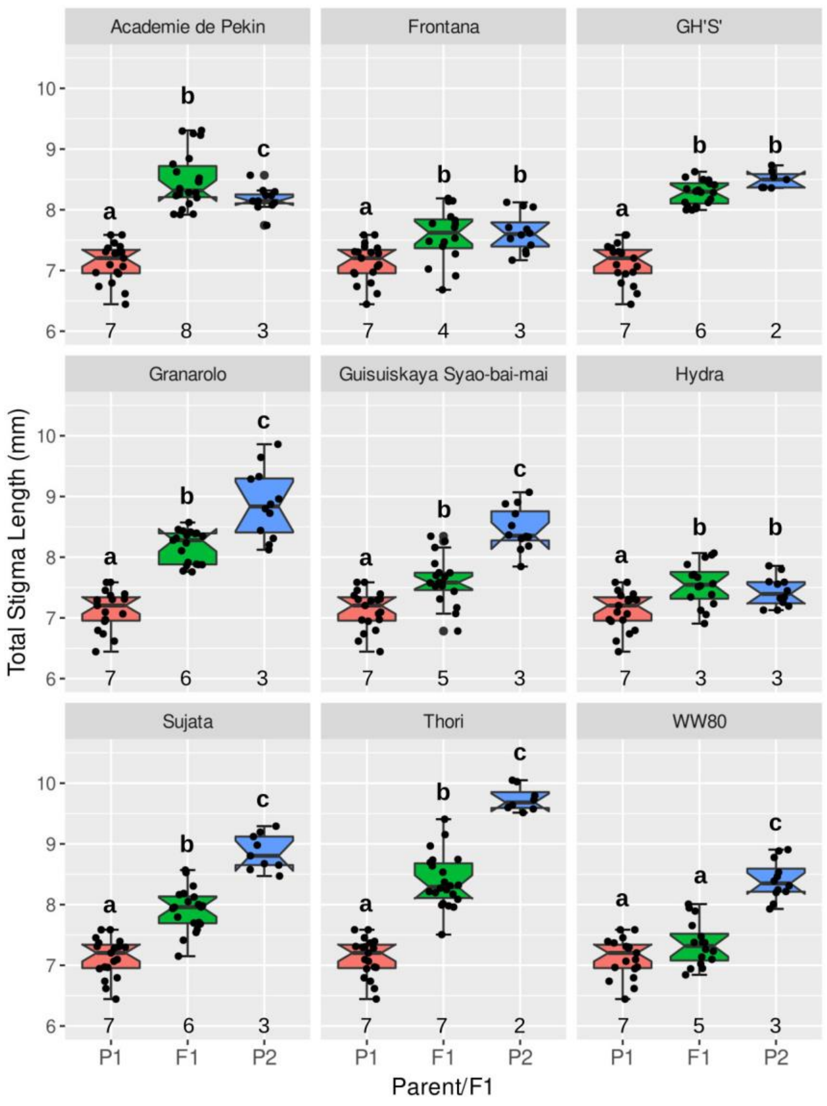

Figure S3. Comparison of TSL in F_1 hybrids and their parents. *Ms3ms3* derivatives of *Ms3*/7*1IBWSN50 were used as female parent (P1) in each case and nine cultivars (indicated on top of each plot) that ranged in TSL value were used as male parents (P2) to generate F_1 lines. Number of plants for each genotype group is indicated at the bottom, jittered dots indicate measurement of stigmas, and groups with different index letters are significantly different based on Tukey's test at $p < 0.05$.

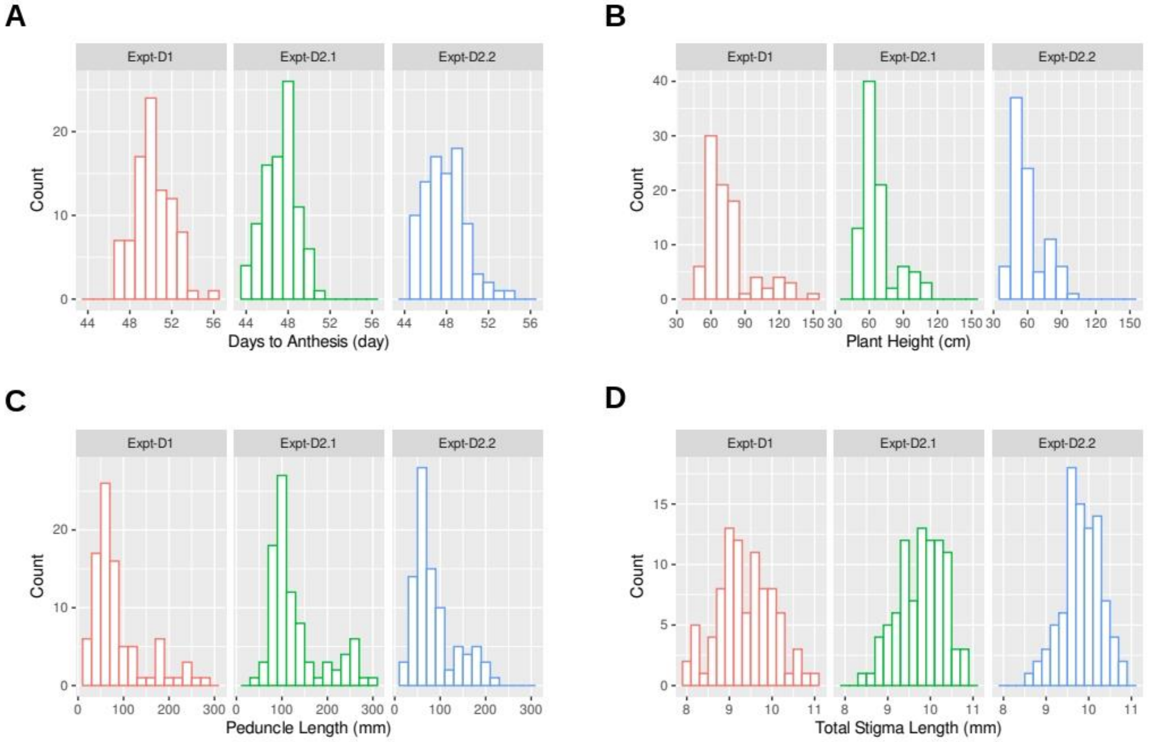

Figure S4. Frequency distributions of traits measured in Mexicali/Italo Expt-D1 and Expt-D2. (**A**) Days to Anthesis. (**B**) Plant Height. (**C**) Peduncle Length. (**D**) Total Stigma Length. Subset of plants of two sowing dates in Expt-D2 are shown in separate histograms as Expt-D2.1 and Expt-D2.2.

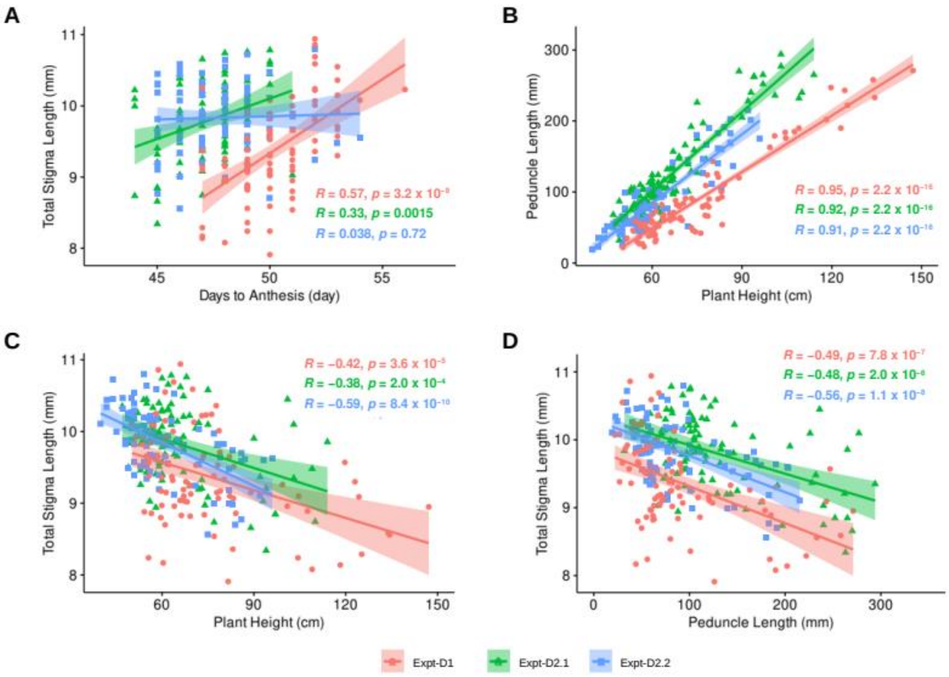

Figure S5. Trait correlation plots for Mexicali/Italo F_2 Expt-D1 and Expt-D2. Data are grouped by sowing date and colour-coded red (Expt-D1), green (Expt-D2.1) and blue (Expt-D2.2). Correlations are shown by scatter plot for (**A**) TSL vs DA, (**B**) PL vs PH, (**C**) TSL vs PH, (**D**) TSL vs PL. Correlation coefficient and *p*-value determined by Pearson's correlation analysis is indicated at the top of each panel. Linear regression lines and 95% confidence intervals (shading) are also indicated.

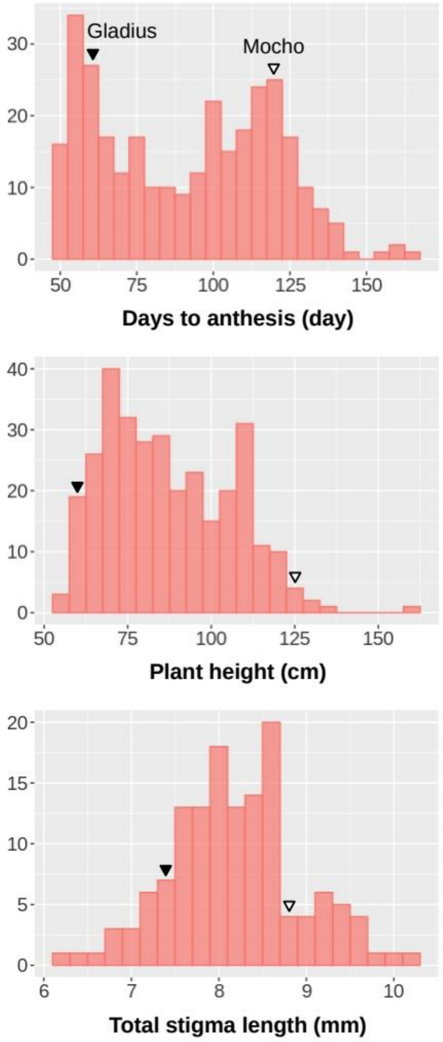

Figure S6. Frequency distributions for traits in the Mocho/Gladius RIL population. Trait names are indicated below each plot. The X-axis indicates trait value, and the Y-axis indicates number of RIL individuals in each class. Measurement classes containing values for parental lines are indicated by open (Mocho) and filled (Gladius) triangles.

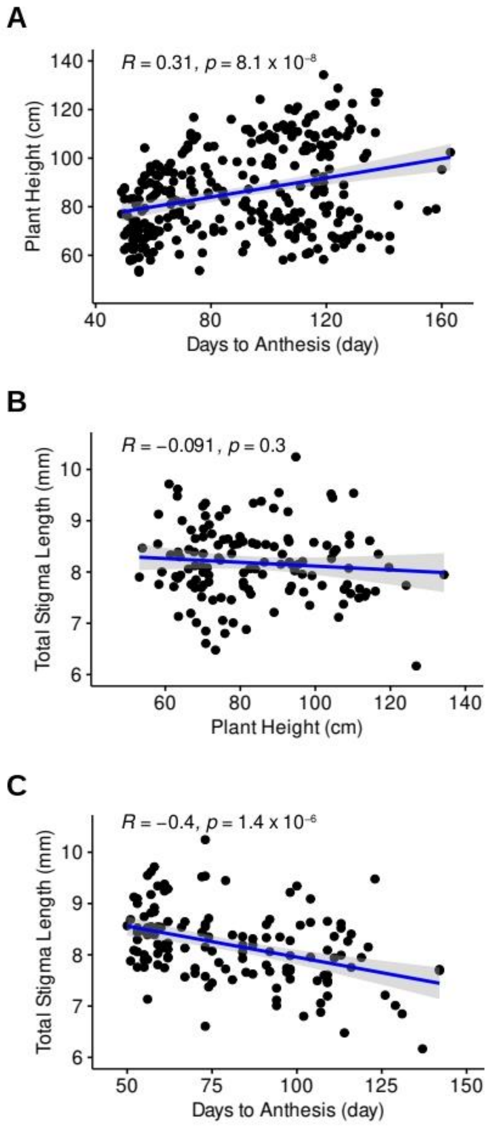

Figure S7. Scatter plots representing correlations between measured traits in the Mocho/Gladius RIL population. (**A**) DA vs PH. (**B**) TSL vs PH. (**C**) TSL vs DA. Correlation coefficient and *p*-value determined by Pearson's correlation analysis is indicated at the top of each panel. A linear regression line (blue) and 95% confidence interval (grey shade) are also indicated.

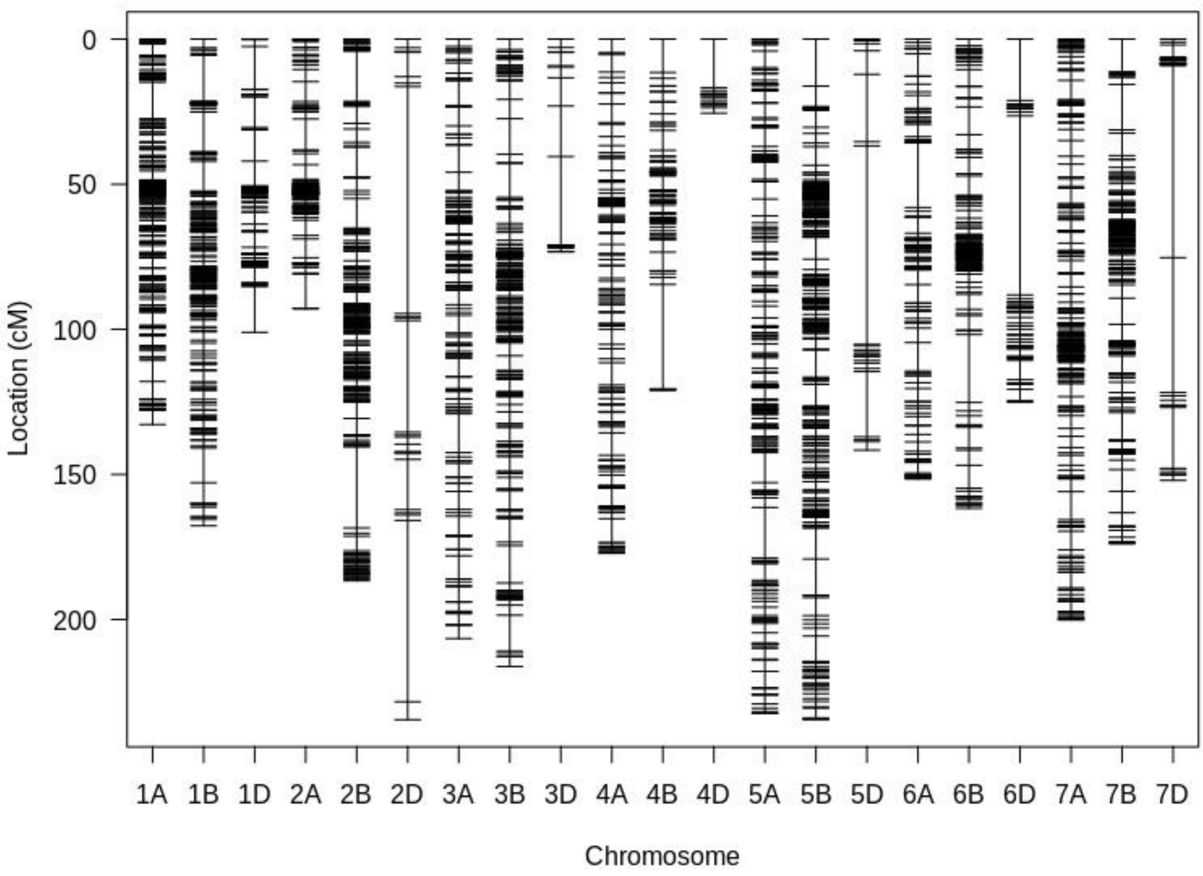

Figure S8. Genetic linkage map for the Mocho/Gladius RIL population. Twenty-one genetic linkage groups were generated and assigned to a wheat chromosome (X-axis). Genetic distance (cM) of the linkage map is indicated on the Y-axis. Marker information for the linkage map is described in Supplementary Table S4.

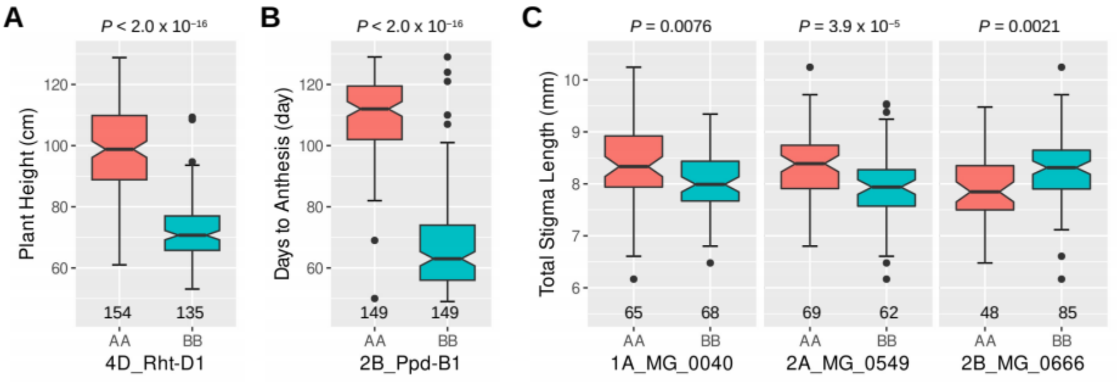

Figure S9. Association of traits with marker genotype at identified QTL in the Mocho/Gladius RIL population. Traits are (**A**) Plant Height, (**B**) Days to Anthesis and (**C**) Total Stigma Length. Boxplots represent trait value (Y-axis) of genotype classes (X-axis) which are shown as follows: AA (red); Mocho-derived, BB (blue); Gladius-derived. Number of RILs in each genotype group is indicated at the bottom. Outliers are indicated by black dots. Significant difference between genotype groups examined by Student's *t*-test is presented as *p*-value above each panel.

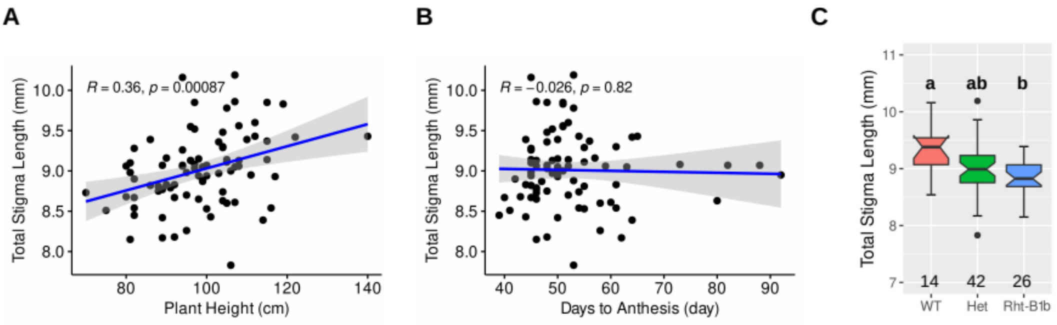

Figure S10. Association of TSL with PH and DA and *Rht-B1* allele effects in the Thori/Hydra F_2 population. (**A**) Correlation of TSL with PH (left panel) and DA (right panel), shown by scatter plot. Correlation coefficient and *p*-value by Pearson's correlation analysis is indicated at the top of each panel. A linear regression line (blue) and 95% confidence interval (grey shade) are also indicated. (**B**) Association of *Rht-B1* genotype with TSL. Homozygous or heterozygous genotypes are indicated on the X-axis as follows: WT; *Rht-B1a* homozygotes (red bar), Het; *Rht-B1b* heterozygotes (green bar), Rht-B1b; semi-dwarf *Rht-B1b* homozygotes (blue bar). Number of plants for each genotype group is indicated at the bottom, and groups with different index letters are significantly different by Tukey's test at $p < 0.05$

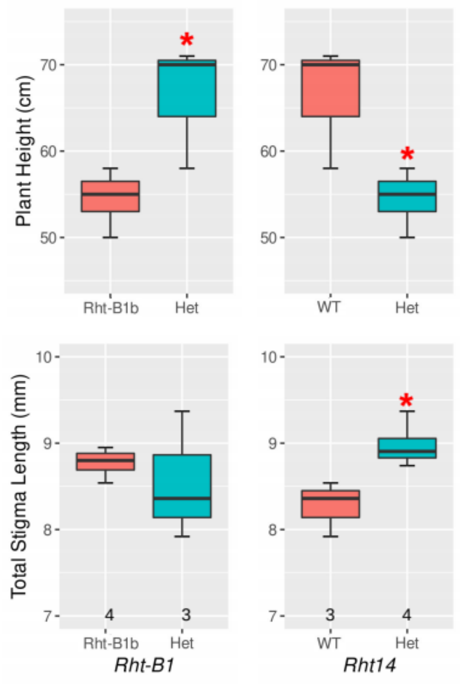

Figure S11. Effect of an Italo-derived 6A locus containing *Rht14* on TSL in bread wheat BC_2F_1 plants. *Rht14* from durum cultivar Italo (*Rht14*, *Rht-B1a*) was introgressed into the bread wheat line *Ms2*/6*SUN276A (*rht14*, *Rht-B1b*). Seven BC_2F_1 plants carrying 2n = 42 chromosomes were used for genotyping and trait measurement. Box plots show PH (top panels) and TSL (bottom panels) values on the Y-axis. On the X-axis *Rht-B1* genotypes are indicated as: Rht-B1b; homozygous *Rht-B1b*, Het; heterozygous *Rht-B1a*/*Rht-B1b*. *Rht14* genotypes are indicated as: WT; homozygous *rht14*/*rht14*, Het; heterozygous *Rht14*/*rht14*. Number of plants in each genotype group is indicated at the bottom, and significant difference identified by Student's *t*-test ($p < 0.05$) between groups is indicated by asterisk.
