# Supplementary material for: Diversity in bread and durum wheat stigma morphology and linkage of increased stigma length to dwarfing gene Rht14
Source: Theor Appl Genet. 2024 Jun 14;137(7):160. doi: 10.1007/s00122-024-04663-4 (PMC11178622; doi:10.1007/s00122-024-04663-4)
Supplement: Supplementary file 2 — Supplementary file2 (PDF 107 kb) [file 122_2024_4663_MOESM2_ESM.pdf]

Method for wheat or barley DNA extraction, without the use of phenol.

96-well format using ball-bearings and freeze-dried samples.

Note: see below for
- **A smaller-scale option that facilitates precipitation directly into storage plates.**
- **A larger-scale option for extraction in 10 ml centrifuge tubes**

Equipment for 96-well extraction

Freeze-dryer
Retsch Mill bead-beater or similar, suitable for 96-well racks of 1.1 ml tubes
Swing-out benchtop centrifuge with holders suitable for 96-well racks of 1.1 ml tubes
65 °C oven
37 °C oven (convenient for removing ethanol from DNA pellets, but not essential)
Multi-channel pipettes, 1200 µl and 200 µl, either 8-channel or 12-channel
96-well racks with lids to hold 96 x 1.1 ml strip tubes
1.1 ml strip tubes (8 tubes per strip) with caps
Stainless steel 3 mm ball bearings
Magnet suitable for removing ball-bearings from tubes
Small esky or portable insulated container
Permanent Texta or similar for labelling purpose
Tweezers suitable for retrieving samples from 1.1ml tubes are handy
Scissors suitable for cutting leaf samples
Ice

Solutions

Extraction Buffer: 600 µl per sample

    0.1 M Tris-HCl pH 7.5
    0.05 M EDTA pH 8.0
    1.25% SDS
    6 M ammonium acetate: 300 µl per sample. Keep stored at 4ºC.
    [Note: Ammonium acetate is highly hydroscopic. When opening a fresh container, use it all and store the prepared solution in a cold room]

Isopropanol (isopropyl alcohol, propan-2-ol): 360 µl per sample
70 % ethanol: 300 µl per sample
R40 Buffer (40 µg/ml RNase A in 1xTE buffer): 35-100 µl per sample

Sample Collection

Notes:
- To avoid mis-positioning of samples during the extraction process, ensure that the 96-well racks are appropriately labelled and that the 12 x 8 strip tubes in each rack are clearly labelled at a prescribed end with a number from 1 – 12 as well as a rack identifier so that strips of tubes will be orientated correctly in the correct rack. Durable Texta or similar is recommended.
- If preferred, ball-bearings can be placed in collection tubes prior to sampling.
  [Alternatively, ball-bearings can be added to tubes after sample freeze-drying and prior to grinding]

- Collection should be timed to allow adequate time for pre-freezing of all samples before they are put into the freeze-dryer. Pre-freezing is achieved by placing the collected samples at -80 °C for a minimum of 2 hours. It can be convenient to collect samples and store them at -80 °C a day, or even several days, prior to the day of freeze-drying.
- Collected samples can be stored at -80 °C for long periods if necessary.
- To minimize DNA degradation, keep collection tubes on ice during the collection process. Placing racks of tubes on top of ice in an esky provides adequate cooling. For best results aim to keep samples cool at all times.
- Leaves should be free of surface water. If plants need watering, do this well in advance of sample collection.
- During collection avoid sap-leakage as much as possible, i.e. try to not squash leaf tissue.
- For cereal crops, leaf tissue from 2-week-old seedlings provides ideal samples, but young tissue from plants of any age is also suitable.
- To achieve consistent DNA yield from all samples, try to collect equal amounts of similarly aged tissue.

Collect approximately 5 cm of leaf, cut into 2 x 2.5 cm sections, into 1.1 ml tubes in a labelled 96-well rack.
Cutting the leaf sample into small pieces that easily slide into the collection tubes is highly recommended.
It is advantageous at the grinding step if all leaf tissue sits well below the rim of the collection tube.

There is no need to cap individual tubes at this stage.
Simply put the rack lids over the racks and secure them with a rubber band or similar.

Freeze-drying

Freeze-dry samples at -20 °C for a minimum of 16 hours, or until fully dehydrated.
Transfer samples directly from -80 °C freezer to freeze-dryer, removing rack lids from the racks before placing racks in the freeze-dryer. Placing racks onto ice for transfer to the freezer-dryer is recommended.
If the 1.1 ml tubes are capped, remove caps from tubes before placing in the freeze-dryer.

After freeze-drying:
If ball-bearings are already in tubes, cap tubes immediately after freeze-drying.
If not already added, add one 3 mm ball bearing to each tube and then place caps on tubes.

Grinding

Notes:

- For best results samples should be ground immediately after freeze-drying. If necessary, dried samples may be stored at room temperature for short periods (e.g., several days) prior to grinding. Longer storage of freeze-dried samples is not recommended as samples will absorb moisture and not grind successfully.
- Check that all of the dried sample is positioned well down in the tubes so that no sample is caught by the tube cap. This will help ensure that all samples are ground successfully.
- Extensive grinding can shear DNA. Keep grinding times to a minimum.
- After grinding, samples can be stored at room temperature or 4 °C for several days if necessary. For long periods, storage at -80 °C is recommended.

Grind samples on bead-beater for 2 minutes at 25 oscillations/second in 1-minute bursts, allowing the samples to cool a little between bursts of grinding.
Check that all samples are being successfully ground as occasionally samples may become compressed into a ball and not grind well.
Samples which fail to grind at this stage can be ground, after extraction buffer has been added, by manual maceration using a suitable device. Caution: take care to avoid cross-contamination between tubes.

Removing ball-bearings from samples

Notes:

- Caution: all ball-bearings must be removed from tubes before any centrifugation step.
- Ball-bearings can be removed either immediately after grinding while the tissue is still dry, or after the extraction step (by use of a magnet).
- Sample loss can be minimised if ball-bearings are removed after the extraction step.
- After removal, ball bearings should be promptly cleaned:

To remove ball-bearings from dry ground tissue:
Taking one strip of 8 tubes at a time, carefully and gently partially invert the strip over a small tray, allowing the ball-bearings to roll out.

To remove ball-bearings from extraction buffer:
Taking one strip of 8 tubes at a time, and using a suitable magnet, gently and carefully raise the ball-bearings out of the 8 tubes. Subtle "jiggling" will free the ball-bearing from sample debris and allow the ball-bearing to be removed with minimal loss of sample.

Cleaning ball-bearings:
Semi-vigorously agitate ball-bearings in 3 changes of RO water and dry on paper towel. A further quick rinse in a small volume of 100% ethanol and then drying on paper towel is optional.

Extraction method

Notes:

- Pre-heat oven to 65 °C
- It may be beneficial to pre-warm Extraction Buffer to 65 °C. This is optional.
- Failure to remove caps from tubes prior to treatment at 65 °C will result in the caps popping off and samples becoming cross-contaminated.

Add 600 µl Extraction Buffer to each tube.

Firmly seal tubes with caps, shake vigorously. Use a plate holder, or something similar, to put over the caps to ensure that they remain firmly in place throughout the agitation.

Remove caps.
These will be re-applied to the tubes so take care to keep them in order and to not cross-contaminate samples.

Cover the rack of opened tubes with a rack cover (or loose plastic bag or piece of cling-wrap) and carefully place in the 65 °C oven. Take care to prevent cross-contamination of samples. Loose covering the tubes reduces evaporation.

Incubate in the 65 °C oven for 30 minutes.

Place rack of tubes at -20 °C for approximately 15 mins to cool down to room temperature.
Alternatively, the rack can be cooled at 4 °C for longer. The aim is to cool, but not freeze, the samples before the cold ammonium acetate is added.

Add 300 µl of cold 6 M ammonium acetate (stored at 4 °C).

Firmly re-apply caps to the tubes.

Shake rack well, again making sure the caps remain firmly in place to avoid loss of sample or cross-contamination.

Incubate at 4 °C for 15 minutes.

Put cover on rack and centrifuge for 15 minutes at 4000 rpm.

Label a fresh box of 12 x 8 strip tubes.

To each of the new tubes, add 360 µl of isopropanol.

Transfer 600 µl of supernatant to the new tube and cap with new lids.

Mix thoroughly and allow DNA to precipitate for 5 minutes at room temperature.

Centrifuge samples for 15 minutes at 4000 rpm to pellet DNA.

Carefully pour off supernatant and drain the tubes by partially inverting onto a small wad of paper towel.
Caution: the pellets may slide out of the tubes, so watch them carefully!

Wash pellets in 200 µl of 70 % ethanol.

Centrifuge again for 1 minute at 4000 rpm so that pellets are positioned firmly at the base of the tubes.

It is advisable to be thorough in removing all ethanol. Residual ethanol may affect downstream processes.
First, remove most of the 70 % ethanol by careful draining.
Then give the samples another short spin so that all the 70 % ethanol collects at the bottom of the tube.
The ethanol can be aspirated it off with a pipet or allowed to evaporate at room temp (or more quickly at 37 °C).
Do not allow the DNA pellet to become completely dry as it may become difficult to rehydrate. Ethanol is sufficiently removed if it is no longer detectable by smell.

Re-suspend the DNA pellet in R40 Buffer. Use 35-70 µl or more, as appropriate for the size of the pellets.
Re-suspension in a larger volume (e.g., 300 - 400 µl) of ultrapure water is also feasible if DNA is to be used immediately. However, this is only recommended if DNA is to be stored for a short period.
Consistent high-quality results are obtained if DNA is stored in a small volume of R40 Buffer and then subsampled and diluted in ultrapure water for use.

Allow the DNA pellet to re-suspend overnight at 4 °C.

After re-suspension, mix the samples by gentle pipetting to evenly distribute the DNA.
Gentle votexing could also be used for this purpose, although, in general, vortexing of DNA may lead to shearing and is not recommended.

It is convenient to transfer samples to 96-well plates for storage. This can be done as part of the DNA-distribution by pipetting step above.

Optional step for samples with high levels of unwanted contaminants:
An additional centrifuge step can be applied after re-suspension of the pellet to remove cellular debris and excess protein. Centrifuge samples for 20 minutes at 4000 rpm and remove most of the supernatant to 96-well plates for storage. In general, this step is not necessary, but it may be considered if problems arise in downstream processes.

Modifications for higher-throughput, smaller-scale extraction with precipitation directly into storage plates

To save time and plasticware, the DNA precipitation step can be performed directly in V-bottom 200 µl 96-well plates. It is important to use V-bottom plates so that pellets sit firmly at the base of the wells.

To achieve reasonable DNA yield in a lower precipitation volume, reduce the volume of Extraction Buffer to 400 µl and the volume of 6 M ammonium acetate to 200 µl.

After the initial centrifugation step, remove 120 µl of supernatant to the V-bottom 96-well plate.

Add 72 µl isopropanol. Allow to precipitate and then centrifuge at 4000 rpm for 15 mins.

After this, the pellets should be firmly enough stuck at the base of the wells that the supernatant can be gently poured off by inverting the plates over a wad of paper towel. Do not tap the plates up and down on the paper towel to remove more supernatant as this could dislodge the pellets. Residual supernatant will be effectively removed in the 70 % ethanol wash step.

To perform the 70 % ethanol wash step, add 100 µl of 70 % ethanol to each well. Allow to sit for a few minutes. Try to not dislodge the pellet.

Centrifuge for 1 minute at 4000 rpm so that pellets are positioned firmly at the base of the V-bottom wells. Tip off supernatant as before. Allow pellets to air-dry either at room temperature or at 65 °C, whichever is convenient. Do not allow to over-dry or the DNA may not re-suspend. When ethanol can no longer be detected by smell, add 15 µl of R40 Buffer to each well. Cover firmly with plate-sealing tape and put at 4 °C overnight to re-suspend. Then add 85 µl of ultrapure water to each well for a final sample volume of 100 µl.

Modifications for larger-scale extraction method in 10 ml tubes

The steps in this method are essentially the same, except that leaf tissue is collected into round-bottom 10 ml centrifuge tubes that are compatible with storage at -80 °C and immersion in liquid N_2.
V-bottom 10 ml centrifuge tubes can also be used if necessary, however tissue grinding may not be quite so effective.

For sample grinding, 2 x 6 mm stainless steel ball-bearings are used per sample.
It is convenient to have these in the tubes at the time of collection.

As above, during collection, samples should be kept on ice.
Collect around 10-15 cm of leaf, fold lengthways so that it fits easily into the 10 ml centrifuge tube.
Loosely folded leaves will grind more easily than tight balls.
Avoid water droplets as these will freeze rock-hard and make grinding difficult.
Avoid release of sap as much as possible, for the same reason.
Cap tubes.

Prior to grinding and extraction, samples should be stored at -80 °C.

Liquid N2 is used for tissue grinding.
Important: When using liquid N_2 wear appropriate PPE and adhere to all safety measures.

Place the capped sample tube, with ball-bearings added, in liquid N_2 and allow to freeze.
Note that tubes should be tightly capped to ensure that no liquid N_2 enters the tube during the freezing step.

Remove the tube from liquid N_2 and immediately slightly loosen the cap so that air can be released. This is critical as otherwise the tube may explode. The cap should not be so loose that it comes off during grinding.

Break up the leaves somewhat by shaking the tube a few times, then hold the tube on a vortex so that the ball-bearings grind the sample. This should be done quickly. Over-processing can shear DNA.
Alternative bursts of hand-shaking and short vortex grinding is most effective.
If young tissue was collected, grinding should only take about 15-30 seconds or so.

After grinding, place the tube on ice to thaw.

When all samples are ready, proceed with the extraction process following the steps outlined above and adjusting volumes appropriately. For example, use 2 ml of Extraction Buffer and 1 ml of 6 M ammonium acetate.

Final re-suspension of DNA pellets should be in R40 Buffer, with pellet size indicating the volume to use (as a guide, this could be somewhere in the range 250 – 400 µl).
